# Rapid evolution of hybrid breakdown following recent divergence with gene flow in *Senecio* species on Mount Etna, Sicily

**DOI:** 10.1038/s41437-022-00576-4

**Published:** 2022-12-09

**Authors:** Edgar L. Y. Wong, Bruno Nevado, Simon J. Hiscock, Dmitry A. Filatov

**Affiliations:** 1 Department of Biology, University of Oxford, Oxford, UK; 2 Senckenberg Biodiversity and Climate Research Centre, Frankfurt am Main, Germany; 3 Centre for Ecology, Evolution and Environmental Changes, University of Lisbon, Lisbon, Portugal; 4 Oxford Botanic Garden and Arboretum, Oxford, UK

**Keywords:** Speciation, Plant evolution

## Abstract

How do nascent species evolve reproductive isolation during speciation with on-going gene flow? How do hybrid lineages become stabilised hybrid species? While commonly used genomic approaches provide an indirect way to identify species incompatibility factors, synthetic hybrids generated from interspecific crosses allow direct pinpointing of phenotypic traits involved in incompatibilities and the traits that are potentially adaptive in hybrid species. Here we report the analysis of phenotypic variation and hybrid breakdown in crosses between closely-related *Senecio aethnensis* and *S. chrysanthemifolius*, and their homoploid hybrid species*, S. squalidus*. The two former species represent a likely case of recent (<200 ky) speciation with gene flow driven by adaptation to contrasting conditions of high- and low-elevations on Mount Etna, Sicily. As these species form viable and fertile hybrids, it remains unclear whether they have started to evolve reproductive incompatibility. Our analysis represents the first study of phenotypic variation and hybrid breakdown involving multiple *Senecio* hybrid families. It revealed wide range of variation in multiple traits, including the traits previously unrecorded in synthetic hybrids. Leaf shape, highly distinct between *S. aethnensis* and *S. chrysanthemifolius*, was extremely variable in F_2_ hybrids, but more consistent in *S. squalidus*. Our study demonstrates that interspecific incompatibilities can evolve rapidly despite on-going gene flow between the species. Further work is necessary to understand the genetic bases of these incompatibilities and their role in speciation with gene flow.

## Introduction

Hybridization has been studied in many species, especially in plants, and was considered an important factor in evolution and speciation by botanists such as G. Ledyard Stebbins (e.g., Anderson and Stebbins, 1954; Stebbins, 1950) and Verne Grant (Grant, 1971). Some biologists argue that it is unlikely for speciation to occur in the presence of gene flow because recombination would break down linkage disequilibrium between differentiated genes and stop divergence from building up (Bolnick and Fitzpatrick, 2007). On the contrary, it has been suggested that hybridisation (gene flow) would not influence the rate of speciation process if divergence is driven by selection in the species with extensive range and narrow hybrid zone, and/ or if the number of migrants between nascent species is low (Abbott et al., 2013; Barton, 2013). Indeed, there are multiple mechanisms by which the impeding effect of recombination can be overcome. For instance, regions under divergent selection would have low effective recombination rate (Via and West, 2008), tight physical linkage (Butlin, 2005), and pleiotropy (Kirkpatrick and Barton, 1997; Kirkpatrick and Ravigné, 2002) that could assist in maintaining divergence in the genes and genomic regions involved. Sometimes, gene flow can even facilitate speciation by increasing genetic diversity that selection can act on (Mallet, 2005; Smadja and Butlin, 2011). Speciation with gene flow has been widely reported in multiple systems, such as butterflies (*Heliconius*: e.g Mallet et al., 2007; Nadeau et al., 2012), equids (*Equus*: e.g., Jónsson et al., 2014), sticklebacks (*Gasterosteus*: e.g., Vines et al., 2016), and monkeyflowers (*Mimulus*: e.g., Ferris et al., 2017; Fishman et al., 2013). As hybrids provide a way to exchange genes between diversified populations and species, they offer valuable opportunities for evolutionary biologists to study the processes involved in evolution of reproductive isolation and species divergence.

If reproductive isolation is present, hybrids would experience selection against them in the form of reduced fitness caused by intrinsic (between hybridising species) and extrinsic (between phenotype and environment) incompatibilities. Selection against hybrids can be manifested in a number of ways, such as failure to germinate or reproduce, growth defects and other extrinsic postzygotic selection (e.g., Moyle and Nakazato, 2008). However, these are hard to detect in a field setting as only plants that survive would be found. Another way to study selection against hybrids is through studying hybrid breakdown, the altered morphology, reduced viability and fertility in hybrids, in a greenhouse setting using synthetic hybrids. In a controlled environment, any reduction in fitness detected would be attributed to intrinsic selection and incompatibilities (selection in the field would likely be higher than that observed in the greenhouse since there would also be extrinsic selection). Many studies have investigated synthetic hybrids between closely-related species to look for hybrid breakdown: low pollen fertility (Martin and Willis, 2010) and chlorosis leading to lethality in hybrids between *Mimulus guttatus* and *M. nasutus* (Zuellig and Sweigart, 2018); increased susceptibility to herbivores in hybrids between *Eucalyptus amygdalina* and *E. risdonii* (Whitham et al., 1994); chlorosis, defective embryo development and others in hybrids between different *Arabidopsis thaliana* accessions (Plötner et al., 2017); poor growth in hybrids between *Oryza sativa* (rice) varieties (Matsubara et al., 2007).

Not only are synthetic hybrids useful to study the potential phenotypic and genetic incompatibilities expressed in hybrids, but they are also useful in studying hybrid speciation through comparisons with natural hybrids. Hybrid speciation is one of the fastest types of speciation (Coyne and Orr, 2004). Young systems of hybrid species are especially valuable, as they allow evolutionary biologists to identify extant progenitor species, and to study the processes leading to hybrid speciation before their signatures get masked by other long-term (such as demographic) processes. By examining generations of synthetic hybrids, the evolutionary processes leading to phenotypic and genomic changes, and their underlying causes which are potentially important for establishing a stable hybrid lineage can be identified. Besides, although there are studies examining the genetic components of hybrid speciation (such as incompatibilities, levels of admixture) (e.g., Schumer et al., 2018; Chaturvedi et al., 2020; Nevado et al., 2020), few studies have explored the morphological aspect of how stable hybrid phenotypes evolve (e.g., Rosenthal et al., 2005) despite the direct role of hybrid morphology in adaptation and survival. Trying to recreate ancient hybrid *Helianthus* species, Rosenthal et al. (2005) found that it was rare for synthetic hybrids to possess the phenotype of the hybrid species; however, the relative abundance of hybrid species phenotypes in synthetic hybrids also depended on the specific lineages. Similar studies have also been done on crop species to investigates changes from hybridisation to domestication (e.g., Zhang et al., 2016). Using synthetic hybrids, some outstanding questions about hybrid speciation could be answered, such as how much of the ecological, morphological and genetic novelty observed in the hybrid species actually came from its progenitors through hybridisation, or whether it is due to divergent evolution in their new habitats after speciation (Rieseberg, 1997).

In this study, we focus on synthetic hybrids and homoploid hybrid species between two *Senecio* species native to Mount Etna, Sicily - *Senecio aethnensis* Jan ex. DC. and *Senecio chrysanthemifolius* Poir. *S. aethnensis* occurs above 2000 m whereas typical *S. chrysanthemifolius* occurs below 1000 m on Mount Etna (Brennan et al., 2009; Muir et al., 2013). The two species maintain a hybrid zone at their intermediate elevations at least on the southern side of the mountain, where they exhibit a range of intermediate phenotypes between the two species (Brennan et al., 2009; Chapman et al., 2005; James and Abbott, 2005). Their divergence as sister-species was estimated to have occurred fewer than 200,000 years ago, coinciding with the rise of Mount Etna to elevations above 2000 m due to volcanic activity (Chapman et al., 2013; Osborne et al., 2013; Osborne et al., 2016). *S. aethnensis* and *S. chrysanthemifolius* are also the progenitor species of *S. squalidus*, a homoploid hybrid species widespread in the UK. It is believed to have originated through hybridisation between *S. aethnensis* and *S. chrysanthemifolius* that were sourced from Mount Etna and subsequently cultivated in British gardens sometime between 1690 to 1710 (Nevado et al., 2020). Previous studies have shown that *S. squalidus* are intermediate both phenotypically and genetically between the two parental species (James and Abbott, 2005; Abbott et al., 2010; Nevado et al., 2020). It is intriguing how quickly *S. squalidus* has stabilised its genome and spread across the UK within merely a few hundred years. The spread of *S. squalidus* was thought to be aided by the establishment of the railway system in the British Isles (Druce, 1927) - herbarium records reveal that the species had reached northern England between early to mid-twentieth century (Abbott et al., 2009) and in the Central Belt of Scotland in the 1950s and 1960s (Kent, 1966). Previous studies have shown that hybrid stabilisation could be achieved with fewer than 60 generations (Rieseberg et al., 1996; Ungerer et al., 1998; Rieseberg, 2000), although more recent modelling showed that stabilisation of the hybrid genome would actually take hundreds of years (Buerkle and Rieseberg, 2008).

*S. aethnensis* and *S. chrysanthemifolius* produce viable and fertile hybrids (Chapman et al., 2005), although natural hybrids on Mount Etna appear to have lower fitness than their progenitors due to both intrinsic and extrinsic selection (Brennan et al., 2014; 2019; Chapman et al., 2016; Wong et al., 2020). Demographic modelling has shown that the two species had most likely experienced substantial gene flow since their divergence (Filatov et al., 2016; Wong et al., 2020), indicating that species incompatibilities are still weak between these *Senecio* species. On the other hand, the reports of hybrid breakdown in F_2_ hybrids (Brennan et al., 2014; Chapman et al., 2016; Hegarty et al., 2009), and evidence of genetic incompatibilities (Brennan et al., 2014; 2016; 2019; Chapman et al., 2016) suggests the two species have evolved some reproductive isolation since their recent divergence.

Brennan et al. (2014) found significant transmission ratio distortion in around 27% of loci studied, some of which exhibited asymmetric differences in transmission ratio distortion due to different parental cytoplasmic background, suggesting potential nucleocytoplasmic incompatibility. Differences in properties in reciprocal hybrids are indicative of nucleocytoplasmic interactions, as they have a similar nuclear genetic background but different cytoplasmic background (cytoplasm is inherited from one parent only) (Levin, 2003). For instance, Leinonen et al. (2011) found that *Arabidopsis lyrata* individuals with local cytoplasmic origins were associated with higher fitness in their local habitats, while Zuellig and Sweigart (2018) found that cytoplasmic differences were the basis of chlorosis observed in *Mimulus* hybrids. Chapman et al. (2016) mapped hybrid breakdown in the *Senecio* hybrids to a few small and one large quantitative trait loci, and also discovered significant transmission ratio distortion in three genomics regions. Using backcrosses between *S. squalidus* and its parental species, Brennan et al. (2019) observed many loci underlying genomic incompatibilities throughout the genome and that previously identified incompatibility loci between the parental species co-located with some of these loci. Strong selection against hybrids on Mount Etna (as high as a 78% drop in fitness) was also reported in Wong et al. (2020). In terms of extrinsic selection, Walter et al. (2020) observed that *S. aethnensis* performed worse in elevations far from its natural range; whereas *S. chrysanthemifolius* only performed worse after winter at the transplant site furthest from its range. Regarding previously reported hybrid breakdown in Mount Etna *Senecio*, 15.9% of F_1_ individuals showed albinism (chlorosis) and germination was as low as 18% in F_3_ (Hegarty et al. 2009); 47.7% of F_2_ individuals showed stunted or necrotic growth in Chapman et al. (2016); 16.7% of F_2_ individuals experienced early mortality, stunted growth or remained vegetative in Brennan et al. (2014). However, Ross (2010) found no signs of hybrid breakdown in the F_2_ hybrids studied. Given these contradictory results, it remains unclear whether the previously reported cases of hybrid breakdown reflect partial interspecific reproductive isolation, or occasional intra-specific incompatibilities. While the above studies used synthetic crosses to detect incompatibility loci and changes to gene expression, the morphological effects of hybridisation in this system, which is an equally important aspect for understanding hybrid speciation, have not been explored yet.

Hence, this study reports a crossing experiment to better investigate the homoploid hybrid speciation of *S. squalidus* and the morphological variability (including potential hybrid breakdown, nucleocytoplasmic incompatibilities and/or maternal effects) evolved in the face of gene flow in the Mount Etna *Senecio* system. Importantly, we generated and analysed multiple crossing families to ensure highly comparable results generated under the same conditions. Previous studies all used one family per study only, which did not allow those studies to test whether the observed effects are specific to the cross or applicable to any crosses between the two *Senecio* species. Furthermore, we compared synthetic hybrids with both wild and greenhouse-grown *Senecio squalidus* to explore the similarities and differences between early-generation and established hybrids. Specifically, we ask: (a) Do all F_2_ hybrids show reduction in fitness, such as survival rate or stunted growth due to hybrid breakdown? (b) Do F_2_ families of different genetic background (different parents) show significant differences in phenotypic traits?; (c) Do F_2_ hybrids which were derived lines of a reciprocal cross show significant differences in properties (are there potential nucleocytoplasmic incompatibilities and/or maternal effects)?; (d) How similar are the traits of synthetic and natural hybrids and what do the results tell us about hybrid speciation in this system?

## Materials and methods

### Crossing design to obtain F_2_ families

**Fig. 1:**
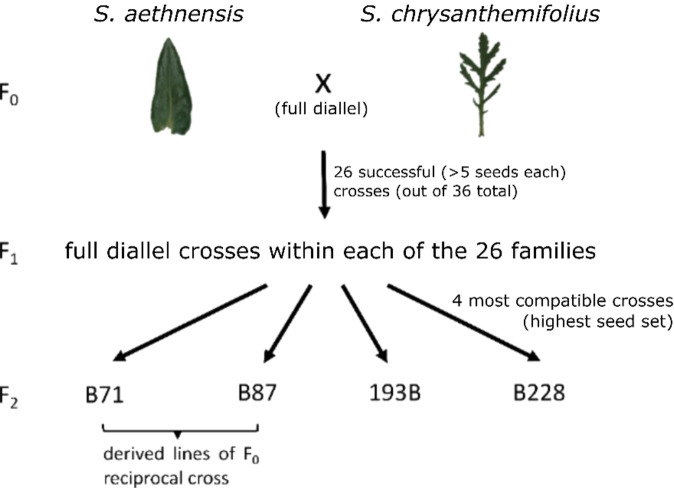
Crossing design in this study. F_0_ parents in 193B, B228, and the derived lines of a reciprocal cross (B71 and B87) were grown from seeds collected from different populations.

*S. aethnensis* and *S. chrysanthemifolius* plants were raised using seeds collected from mature plants of wild populations on Mount Etna. *S. aethnensis* seeds were collected from populations between 2500 and 2600 meters above sea level (masl), while *S. chrysanthemifolius* seeds were collected between 730 and 790 masl (Table 1). They are assumed to be pure species as the seeds were collected from plants that were morphologically typical of either species and from the species’ typical range. However, admixture cannot be completely ruled out. Mature plants of both species, and later F_1_ individuals derived from the same parental cross (full siblings) were crossed in a full diallel design (Fig. 1). In F_0_, 12 *S. aethnensis* and 21 *S. chrysanthemifolius* seeds and plants were grown (100% germination and survival), creating 26 (out of 36) F_1_ families with different parental combinations upon successful fertilisation and each with at least five seeds (Fig. 1). Two to eight F_1_ individuals survived to reproductive maturity in each F_1_ family. Individuals within the same F_1_ family were then crossed, generating multiple F_2_ families (each with the same male and female F_1_ parents). The four F_1_ crosses with the highest seed set (to ensure sufficient samples for studying within family variation and prevalence of any breakdown syndrome), including two which were derived from a reciprocal parental F_0_ cross, were chosen to be the F_2_ families of focus in this study. Members of each F_2_ family were offspring of crosses between a pair of full siblings (details of F_0_ and F_1_ parents in Table 1; Fig. 1). This design allows the maternal and paternal origins of each F_2_ and F_1_ individual to be recorded for testing of potential nucleocytoplasmic or maternal effects. Previous studies did not find any evidence of inbreeding depression in inbred F_2_ plants – there was no excess of heterozygotes (the opposite would be true if inbreeding depression was present due to expression of many deleterious homozygotes; Brennan et al., 2014; 2019). Thus, in the current study, we assume that the abnormal phenotypes in F_2_ were due to hybrid breakdown rather than inbreeding depression.

**Table 1:** Parental Information and germination and survival of each F_2_ family used in this study.

F_2_ family	F_0_ ♂ parent (pure species)				F_0_ ♀ parent (pure species)			
	Species	Collected from			Species	Collected from		
		Elevation (masl)	Population	Plant ID		Elevation (masl)	Population	Plant ID
B71	*S. aeth*	2500	2103	3	*S. chry*	790	2097	3
B87	*S. chry*	790	2097	3	*S. aeth*	2500	2103	3
193B	*S.chry*	730	2100	1	*S. aeth*	2500	2104	1
B228	*S. aeth*	2600	2106	1	*S. chry*	790	2098	3

F_2_ family	F_1_ plants (full siblings in each F_1_ family)				F_2_ plants (offspring from crosses among full silbings)						
	F_1_ family no.	No. seeds sown	No. germinated & survival	% germination & survival	No. seeds sown	No. germination	% germination	No. survival	% survival	No. early mortalities	% early mortality
B71	AC02	10	6	60	64	39	60.94	39	60.94	0	0
B87	AC03	10	6	60	59	31	52.54	30	50.85	1	1.69
193B	AC15	10	4	40	50	50	100	39	78	11	22
B228	AC20	10	6	60	64	40	62.5	40	62.5	0	0

Each F_2_ family has known F_0_ and F_1_ maternal and paternal individuals: plants in the same F_1_ family were full siblings, and F_2_ plants were created by crossing full siblings in each F_1_ family. F_0_ parental individuals were grown from seeds collected from different populations (except for those in the reciprocal cross in family B71 and B87). See Fig. 1 for crossing design.

For the final four F_2_ families that were chosen in this study, each had F_0_ parents collected from different populations on Mount Etna, except the two derived lines of a F_0_ reciprocal cross (where they shared the same parental individuals but opposite crossing direction). In other words, F_0_ parents of each of the non-reciprocal F_2_ families were independent of one another.

### Seed germination and plant-rearing conditions

All germination and rearing were carried out in temperature-controlled glasshouses with a 16-hour photo period (LED lighting) at 20°C. Seeds were germinated in soil mixture comprising 3:1 mixture of compost and medium grade vermiculite, in standard 8 × 5 tray pots. Seedlings were transplanted to 12 cm pots once they were established, using soil mix of the same composition as that for germination. Pots were randomised weekly.

To cross individuals, mature capitula were rubbed together, enclosed with cellulose mesh bags then tied. Each bunch of flower heads were enclosed with cling film packets before artificial fertilisation to avoid any unrecorded wind pollination. The two *Senecio* species are highly self-incompatible (Chapman et al., 2005; Brennan et al., 2013) thus there was no need to emasculate the capitula before crossing. Hence all crosses were reciprocal as each capitulum was both the donor and recipient of pollen.

### Sampling of *Senecio squalidus*

Wild *S. squalidus* leaves were collected from four locations in the United Kingdom—Oxford, Exeter, Newcastle, and Edinburgh, to cover different locations of the species’ range. Two to four mid-stem leaves in good conditions were collected from each individual and photographed on gridded paper for subsequent analyses. Only undamaged leaves were collected from the main stem of the plants, and different sizes of leaves were collected to represent the average leaf of each plant. All plants used in leaf collection were in their reproductive stage. Plants grown in the wild likely have limited resources and more variable growth conditions than those grown in the greenhouse, leading to overestimation of differences between F_2_ plants and *S. squalidus*. Hence, a subset of the wild *S. squalidus* cuttings from Exeter were moved to the lab and grown in the same greenhouse conditions as the F_2_ plants. These greenhouse grown *S. squalidus* plants were compared with both their wild counterparts and F_2_. No floral measurements were taken from any of the *S. squalidus* samples as capitula would be of different floral stages in the wild samples and from plants of an older age than the greenhouse samples.

### Phenotypic characterisation

At the first sign of flower buds in each F_0_, F_1_ and F_2_ individual, three mid-stem leaves were randomly chosen and photographed on gridded paper. Leaf images were scaled individually and analysed in *LeafProcessor* (Backhaus et al., 2010). Various leaf morphometric parameters were estimated by the software, including perimeter, area, length, width, compactness (ratio of perimeter squared and area). Leaf phenotypes were chosen because leaf dissection remains the most characterising feature in the target *Senecio* groups.

Various other phenotypic traits were measured in F_2_ individuals manually with the aim to explore trait variations and identify any potential signs of hybrid breakdown in F_2_ individuals grown in the greenhouse (sample sizes of F_1_ and F_0_ parents for each F_2_ family were too small for meaningful comparisons). These include presence of trichomes on leaves, plant height at first anthesis (cm), number of effective branches (branches with two or more nodes) at first anthesis, stem diameter at first anthesis (mm), number of ray flowers on apical capitulum, number of capitula on the apical umbel, width of receptacle of apical capitulum (disc diameter; mm), and length of pedicel of apical capitulum (mm).

### Statistical analyses

In the leaf measurements, entries with leaf area below 3 or above 400 cm^2^ were outliers and treated as measurement error and removed from the dataset. Where analyses only involved F_2_ individuals (both leaf and other traits), measurements were averaged among the three leaves measured for each individual (as there was only one entry per individual for other traits). In leaf trait-only analyses, all leaf measurements were used as separate entries.

First, principal component analyses (PCA) were carried out using the R package *ggbiplot* (Vu, 2011) to visualise: (a) whether F_2_ individuals are intermediates between and their F_0_ and F_1_ progenitors, and F_1_ between their F_0_ progenitors; (b) how different overall the four F_2_ families (and the two which were derived lines of a reciprocal F_0_ cross) are; (c) how different F_2_ individuals are compared to the wild *S. squalidus*; and (d) how different F_2_ individuals and *S. squalidus* from Exeter (collected in Exeter or grown from cuttings from Exeter) are; (e) how different F_2_ individuals are compared to their F_1_ parents in each F_2_ family. Non-metric multi-dimensional scaling (NMDS) was carried out for (c) and (d) as an alternative clustering approach to test the differences between groupings, using the R package *vegan* (Oksanen et al., 2013). For all of these analyses, data involving ratios (leaf length: width, leaf perimeter:area, and leaf compactness) were excluded to avoid the artefacts of multicollinearity.

Summary statistics, including mean, standard deviation, minimum and maximum values, of all traits were obtained using the R package *psych* (Revelle, 2019), in each of the F_2_ families and all F_2_ individuals and *S. squalidus*. To test whether the differences are significant in groups (a) and (b) above, a non-parametric test of multivariate analysis of variance (MANOVA) was carried out using the R package *npmv* (Ellis et al., 2017), using PC scores from PCA analyses. For the groups with NMDS plots [groups (d) and (e)], the non-parametric analysis of similarity (ANOSIM) test was carried out with the R package *vegan* (Oksanen et al., 2013), instead of MANOVA. Significant differences (especially in breakdown traits such as number of ray florets) between F_2_ and *S. squalidus* would indicate the presence of hybrid breakdown (and adaptation of the latter to the UK habitats for certain traits related to growth), while significant differences among F_2_ families would indicate varying levels of differentiation and incompatibility (e.g., if only some families showed early mortality) between the F_0_ parents. If there are significant differences between the F_2_ families derived from the F_0_ reciprocal cross, this would suggest a potential nucleocytoplasmic or maternal effect on phenotypes.

To further test which traits are significantly different and correlated among and between each pair of the four F_2_ families, and between F_2_ and *S. squalidus*, Kruskal-Wallis test, Wilcoxon rank-sum tests were carried out using the R package *stats* (R Core Team, 2013); and Spearman’s correlation tests were carried out for all traits except leaf perimeter:area, length:width and compactness, using the R package *corrplot* (Wei and Simko, 2021). This is to identify traits with potential hybrid breakdown and those that would be affected by different genetic backgrounds, which would point to varying levels of genetic incompatibility between parental species (evidence for incomplete reproductive isolation) and potential nucleocytoplasmic incompatibility. The same tests were also done between wild *S. squalidus* samples from Exeter and greenhouse plants grown from cuttings of these individuals, to test the effect of a controlled environment on the phenotypes.

## Results

### Early signs of hybrid breakdown

**Fig. 2:**
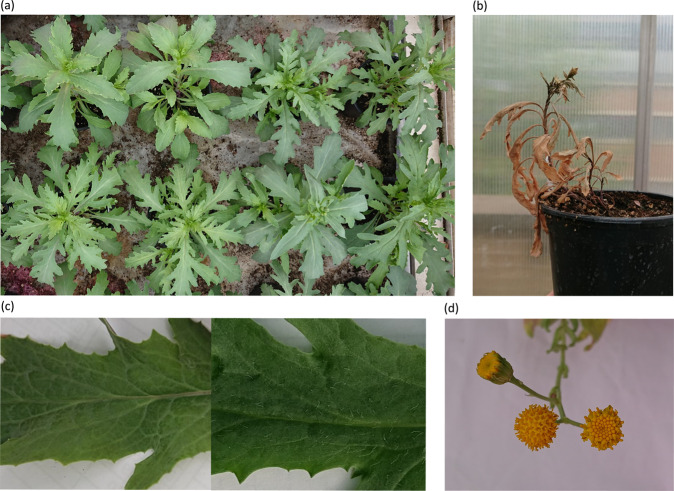
Photos showing trait variabilities and potential signs of hybrid breakdown. **a** Leaf shape variation in the same F_2_ family; **b** A F_2_ plant showing early mortality; **c** Left: leaf without trichomes (parental phenotype); right: F_2_ with trichomes; **d** F_2_ capitula that lack ray florets.

The number of individuals that germinated and survived to maturity was variable among the four F_2_ families, ranging from 52.54% to 100% for germination and 50.85% to 78% for survival (Table 1). Two out of four F_2_ families showed early mortality (Fig. 2)—one (1.69%) and 11 (22%) individuals in families B87 and 193B respectively (Table 1). These individuals all grew to maturity and had flower buds before they died. However, they either died before the flower buds opened or shortly after they started flowering (before they could set seeds). Other signs of hybrid breakdown were also present in F_2_ individuals, including capitula that lacked ray florets (Fig. 2). However, other previously reported signs of hybrid breakdown, including plants remaining vegetative, necrosis, and albinism were not observed in the hybrids in this study.

F_1_ individuals grown in this experiment did not show obvious hybrid breakdown traits regarding phenotypes (e.g., lack of ray florets, low germination, low survival and early mortality in F_2_), except that one F_1_ family (out of 26 that were sown) had individuals with capitula that lacked ray florets (this hybrid line was not used in this study as there were insufficient seeds for growing F_2_).

### Significant phenotypic differences between F_2_ hybrids and *S. squalidus*

**Fig. 3:**
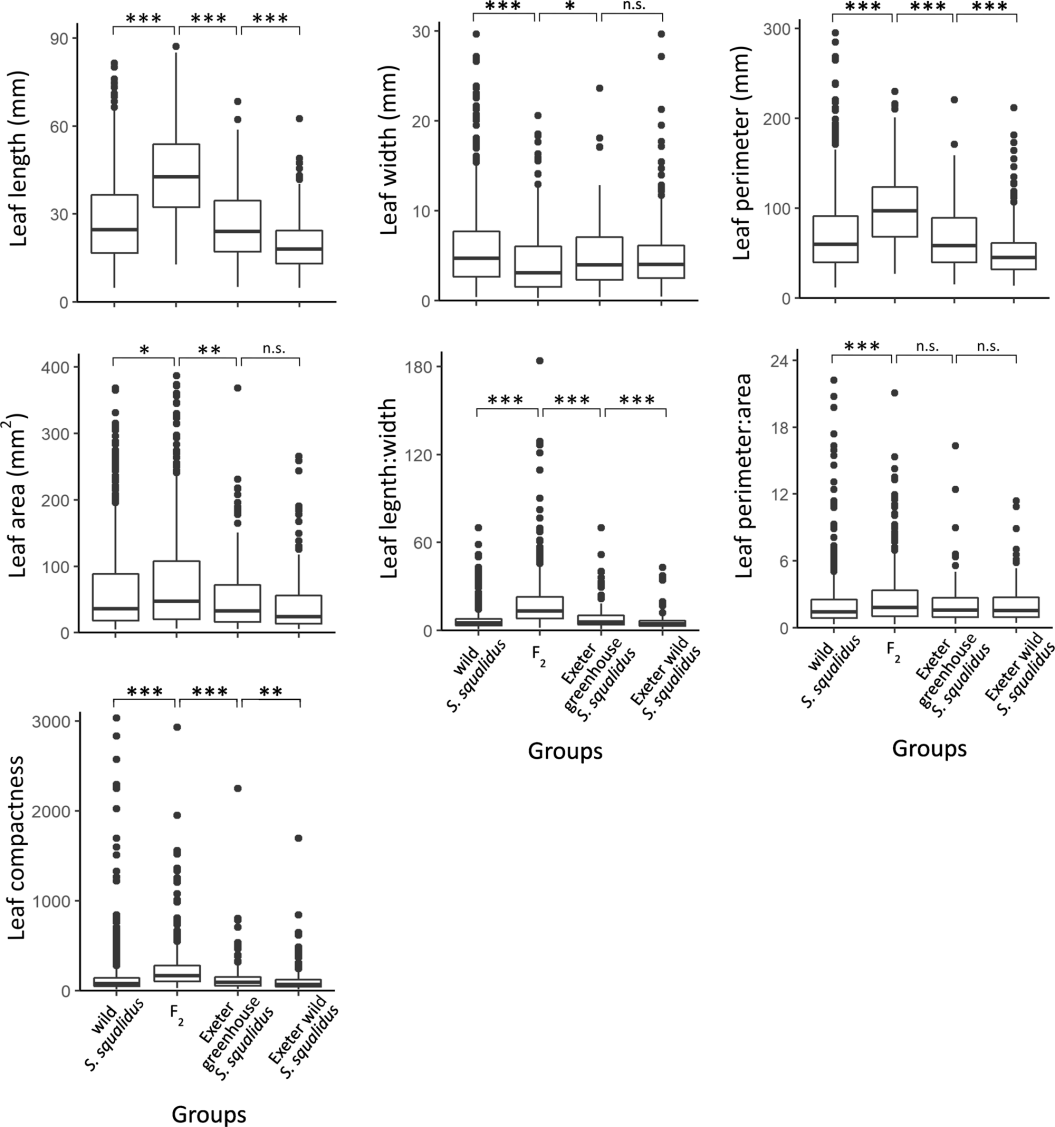
Box plots of each leaf phenotypic trait quantified in wild *S. squalidus*, F_2_ individuals, greenhouse-grown and wild Exeter *S. squalidus* samples respectively. * indicates significance of each pairwise Kruskal-Wallis test (*: *P*-value < 0.05; **: *P*-value < 0.005; ***: *P*-value < 0.0005; n.s. = not significant).

**Fig. 4:**
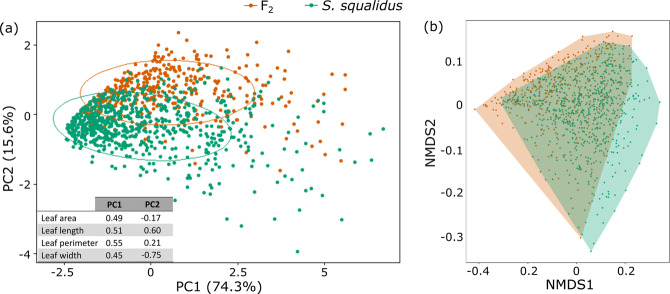
Ordination plots of F_2_ individuals and all wild *S. squalidus* samples. **a** PCA. Ellipses represent 95% CI of the data; **b** NMDS plot. Polygon encloses all data points. Loading values for PC1 and PC2 are embedded in plot (**a**). Full tables of loading values are in Supplementary Table 5.

A total of 583 wild *S. squalidus* leaves were analysed. All leaf traits (width, length, perimeter and area) were significantly correlated with each other (Supplementary Fig. 1). These traits were then compared with the F_2_ hybrids. Non-parametric ANOSIM test showed that F_2_ and wild *S. squalidus* were significantly different (*p*-value = 0.0001; Supplementary Table 1). Statistics of leaf traits of all *S. squalidus* and F_2_ individuals are summarised (Supplementary Table 2). *S. squalidus* and F_2_ hybrids were significantly different in all traits (Supplementary Table 3). *S. squalidus* had significantly shorter leaves, wider leaves, shorter perimeter, smaller area, smaller length:width, smaller perimeter:area and lower compactness than F_2_ hybrids (Fig. 3, Supplementary Table 3). Although *S. squalidus* and F_2_ hybrids were significantly different overall with several significantly different traits, they showed some degree of overlapping in clustering analyses (PCA and NMDS) (Fig. 4).

**Fig. 5:**
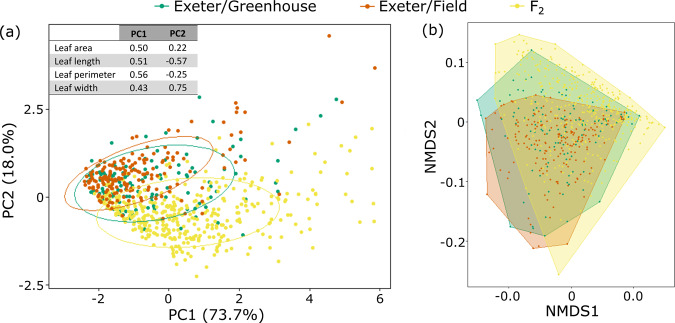
Ordination plots of (1) F_2_ individuals, the hybrid species *S. squalidus* Exeter population that were (2) collected from the field and (3) grown from wild cuttings in the greenhouse. **a** PCA. Ellipses represent 95% CI of the data; **b** NMDS plot. Polygon encloses all data points. Loading values for PC1 and PC2 are embedded in plot (**a**). Full tables of loading values are in Supplementary Table 5.

In the second group involving *S. squalidus* plants grown from cuttings collected from Exeter, 124 greenhouse *S. squalidus* leaves were analysed. Non-parametric ANOSIM test showed that they were also significantly different from F_2_ individuals grown in the greenhouse (*p*-value = 0.0004; Supplementary Table 1). Statistics of leaf traits of these two groups are summarised in Supplementary Table 2. The two groups were significantly different in all traits except leaf perimeter:area (Supplementary Table 3). F_2_ individuals had significantly longer leaves, narrower leaves, longer leaf perimeter, larger area, larger length:width and higher compactness (Fig. 3). In the test of whether the greenhouse environment has a significant effect on the phenotypes, ANOSIM test showed that the greenhouse and wild Exeter samples were significantly different (*p*-value = 0.0012; Supplementary Table 1). Greenhouse-grown samples were significantly different from their identical counterparts in the wild for leaf length, leaf perimeter, leaf length: width and leaf compactness; but not leaf width, leaf area, and leaf permeter:area (Fig. 3). PCA also did not show clear clustering of greenhouse and wild samples (Fig. 5). Clustering analyses showed that Exeter samples in the greenhouse mostly overlap with field samples but with a few outliers (Fig. 5). Although many F_2_ hybrid samples overlap with both types of Exeter samples, plenty of F_2_ samples sit outside of the ranges of the Exeter samples (Fig. 5).

### Significant differences among F_2_ families

**Fig. 6:**
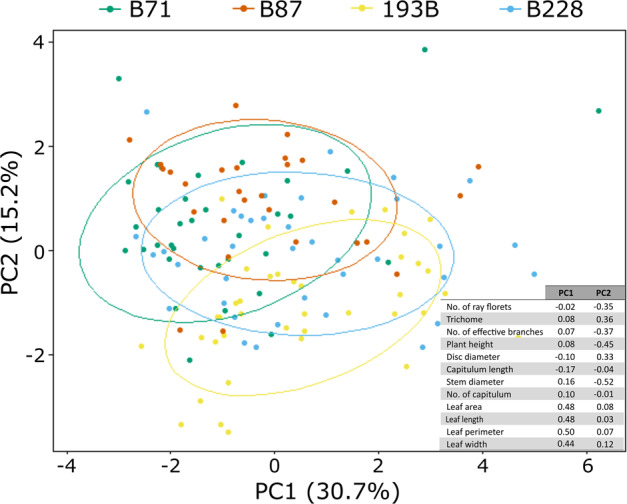
Ordination plot (PCA) of the four F_2_ families. Ellipses represent 95% CI of the data. Loading values for PC1 and PC2 are embedded in the plots. Full tables of loading values are in Supplementary Table 5.

**Fig. 7:**
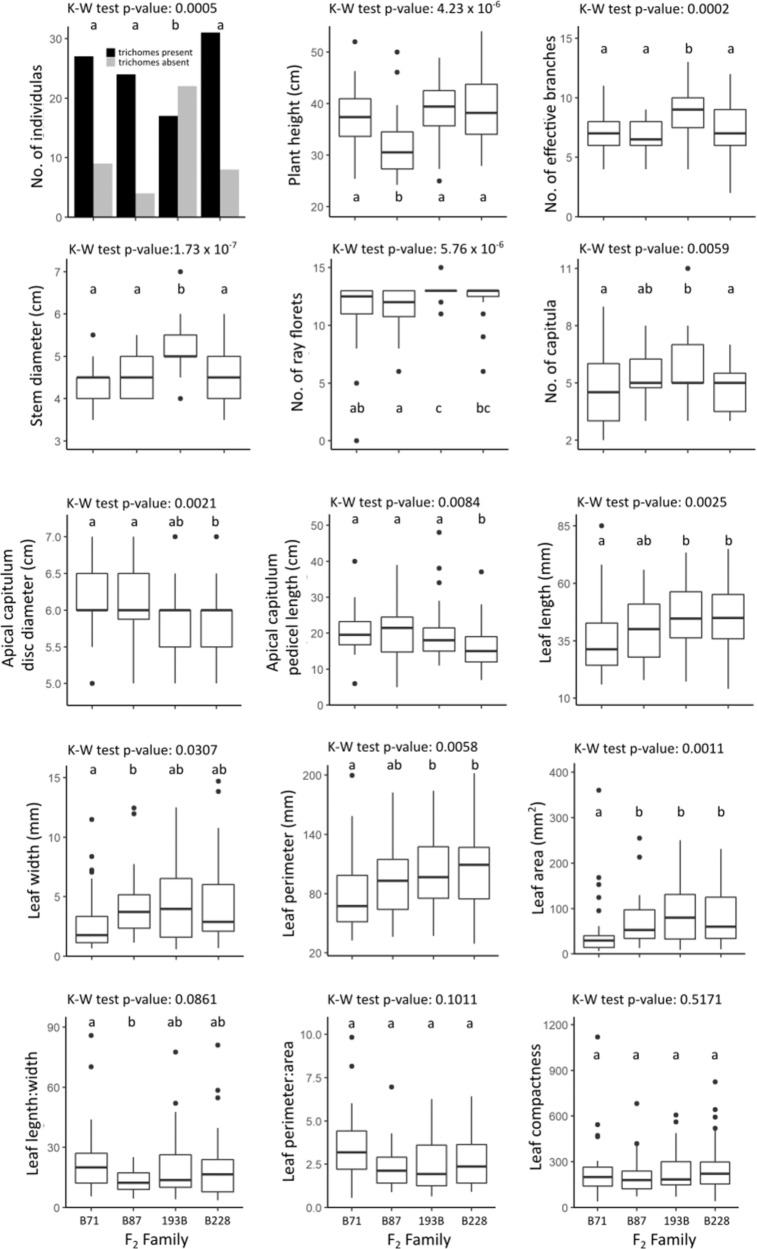
Box plots of each phenotypic trait quantified in the four F_2_ families. *P*-values for Kruskal-Wallis test are indicated at the top of each plot; letters above each box represent groupings following results of Wilcoxon rank-sum test. Detailed results of the two tests can be found in the supplementary tables.

Clustering analyses showed that F_1_ and F_2_ hybrids are clearly intermediate between the two pure species on one PCA axis, while showing more variation in another (Supplementary Fig. 3). *S. chrysanthemifolius* also seems to show more variation than *S. aethnensis* samples (Supplementary Fig. 3). However, F_2_ families, were not distinct from one another (Fig. 6). MANOVA revealed that traits were significantly different among the different generations and the four F_2_ families (p-value < 0.0001; Supplementary Table 1; summary statistics of traits in Supplementary Table 2). In particular, all traits were significantly different among the four F_2_ families (Fig. 7, Supplementary Table 3). It was further revealed that different family pairs had significantly different trait measurements (Fig. 7, Supplementary Table 2). For instance, family 193B had significantly fewer individuals with leaf trichomes, more effective branches (branches with two or more nodes), and larger stem diameter than the other three families; family B71 had significantly smaller leaf area than the other three families; family B87 individuals were significantly shorter than the other three families.

Spearman’s correlation analysis (Supplementary Fig. 2) showed that among the floral traits (number of ray florets, number of capitula, apical capitulum disc diameter, apical capitulum pedicel length), only the number of capitula was negatively correlated with apical capitulum pedicel length. All other comparisons were not significantly correlated. Among the leaf traits (length, width, perimeter, area), all pairs were significantly correlated positively or negatively, except for width and perimeter respectively. Among other vegetative traits (plant height, number of effective branches and stem diameter), stem diameter was significantly correlated with the other two traits, but the number of effective branches was not correlated with plant height. Some traits regarding size were also significantly but negatively correlated with each other, such as stem diameter with apical capitulum disc diameter, leaf length or leaf area.

Clustering analyses of each F_2_ family with their respective F_1_ parents showed that two of the families’ samples overlap with both of their respective parents (B71 and B193), whereas the other two’s samples resembled one of their respective parents more than the other (B87 and B228) (Supplementary Fig. 4, trait values in Supplementary Table 2). Similar to clustering analyses among different generations of parents and hybrids (Supplementary Fig. 3), samples in each F_2_ family showed greater variation outside of their respective parental ranges (Supplementary Fig. 4).

### Significant differences between F_2_ derived from reciprocal F_0_ cross

Non-parametric MANOVA revealed that traits were significantly different between F_2_ families B71 and B87 (*p*-value = 0.0001; Supplementary Table 1). It was further revealed that these two families only had four significantly different trait measurements, among those none were floral traits (Fig. 7, Supplementary Table 4). Family B71 was significantly taller, had significantly narrower leaf width, smaller area, and higher leaf length:width. Clustering analyses also showed large overlap between samples of the two families (Fig. 6).

### Variance of trait values among natural hybrids, synthetic hybrids and their parents

Based on different clustering analyses, F_2_ hybrids consistently occupy more space than F_1_ and F_0_ plants (Supplementary Fig. 3). They occupy similar space as *S. squalidus* overall (Fig. 4), but more space than *S. squalidus* samples from Exeter (Fig. 5). Based on standard deviations (√variance) of each trait (Supplementary Table 1), F_2_ hybrids showed more variance than the natural hybrids, *S. squalidus*, in four of seven leaf traits: leaf area, length, length:width, and perimeter:area; but not for leaf perimeter, width and compactness. F_2_ hybrids’ variances in all traits (area, length, perimeter, width) are in between those of F_1_ hybrids and *S. squalidus*. For perimeter:area, which is a proxy for leaf dissection and a main distinguishing feature between the progenitor species, *S. squalidus* and F_1_ hybrids’ variance is in between that of the two pure species, while F_2_ hybrids have the greatest variance among all groups. For *S. squalidus*, its trait variances are either closest to those of both pure parental species (when the two are on the same end of the spectrum; e.g., leaf area) or one of them (when the two are on opposite ends of the spectrum; e.g., leaf perimeter:area).

## Discussion

Unlike previous studies that only focused on one cross family each that showed the presence or absence of hybrid breakdown (Brennan et al., 2014; Chapman et al., 2016; Hegarty et al., 2009; Ross, 2010), the multiple families of synthetic hybrids used in this study suggested that the presence of new (lack of ray florets) and previously reported breakdown traits (low germination and survival rate) are not cross-specific. Different F_2_ hybrid families showed significant trait differences overall and were significantly different from the natural hybrid *S. squalidus* although leaf trichomes unique to *S. squalidus* were recreated in early-generation synthetic hybrids. Significant differences between the pair of reciprocal crosses provided evidence for potential nucleocytoplasmic incompatibilities and/or maternal effects. Genetically identical *S. squalidus* collected from the field and grown in the greenhouse had significant differences in overall morphology but not in leaf dissection (perimeter:area), suggesting some consistency in leaf shape.

Given that *S. aethnensis* and *S. chrysanthemifolius* represent a likely case of speciation with gene flow (Filatov et al., 2016; Wong et al., 2020), results of this study suggest that hybrid breakdown and its underlying incompatibilities could evolve very quickly despite ongoing gene flow. It also suggest that stabilisation of hybrid lineages, at least at the phenotypic level, can evolve rapidly.

### Various traits leading to low fitness in hybrids

Survival of hybrids is a direct measure of fitness. In this study it was even lower than previously reported: 40–60% in F_1_ and as low as 50.85% in F_2_ hybrids were reported in this study (Table 1), compared to 94% and 73% in F_1_ and F_2_ respectively in Hegarty et al. (2009). In contrast, F_0_ seeds all germinated and grew to maturity for crossing experiments.

Some of the significant trait differences among hybrid families will also likely lead to different fitness levels of the hybrids (Figs. 4, 7). For example, as cues and carrier of rewards for pollinators, different number of ray florets and capitula will directly affect visitations and reproductive success. A smaller size and fewer branches would also affect the number of capitula that could be produced. Low fitness in early generation hybrids is expected, as they harbour the most unfavourable association of alleles compared to later generation ones (Arnold and Bennett, 1993; Fishman and Willis, 2001; Johansen-Morris and Latta, 2006). New signs of hybrid breakdown - the lack of ray florets on capitula was also reported in our study (Fig. 2). Together with the fact that some of the F_2_ families have shown early mortality, these show that that genetic incompatibilities would manifest phenotypically in the system as well.

It is worth noting that this study may have artificially selected for families with higher fertility (with large number of seeds), which is true for most studies on synthetic hybrids (e.g., Abbott and Brennan, 2014). Previous studies only started with one initial parental cross (Brennan et al., 2014; Chapman et al., 2016; Hegarty et al., 2009), while this study started with 36 F_0_ parental crosses, then followed by 26 F_1_ crosses, and focusing on the four crosses with the most number of seeds for phenotypic measurements. Although seed set in F_0_ and F_1_ was not systematically quantified in our study, many of the F_1_ families we generated had very few, if any, seeds and our analysis of F_2_ generations focused on four families with the largest number of seeds available. Hence our results likely underestimated the overall level of hybrid breakdown. Furthermore, so far only the study of Hegarty et al. (2009) has investigated hybrids past the second generation. Hence, more evidence is needed to see whether and how much hybrid fitness could restore in later generations.

### Extent of hybrid breakdown and hybrid phenotypes are likely dependent on genetic background

The observed variation in F_2_ phenotypes was intermediate between the two parental species overall. They are expected to have mixed, reduced fitness on Mount Etna, as trait measures follow a clinal pattern on the mountain which suggests the presence of extrinsic selection acting on phenotypes (Brennan et al., 2009; Wong et al., 2020). Together with other breakdown traits such as low germination, early mortality, albinism and necrosis observed in this and previous studies (Brennan et al., 2014; Chapman et al., 2016; Hegarty et al., 2009), it is unequivocal that hybrid breakdown is present in Mount Etna hybrids. However, the severity of breakdown seems to be dependent of genetic background (e.g., in this study two of the F_2_ families did not s how early mortality), as suggested in other study systems (e.g., Rosenthal et al., 2005). *S. aethnensis* and *S. chrysanthemifolius* individuals that have fewer incompatibilities among them could create hybrids and subsequent offspring that perform relatively better (such as ones that would not suffer from early mortality).

Apart from hybrid breakdown, variation seen in the F_2_ families could also be due to parental differences. The crosses in this study produced many fertile hybrids, which is somewhat contradictory to the low abundance of hybrids (many of which are subtle introgressed forms of each species based on morphology such as leaf shape; personal observations) at intermediate altitudes on Mount Etna and our previous work that revealed strong cumulative selection against hybrids in the wild (Wong et al., 2020). This could also be due to low availability of suitable habitats for hybrids at intermediate elevations. Besides, the observed clinal pattern for leaf shape on Mount Etna (Brennan et al., 2009; Wong et al., 2020) suggests that inconsistent leaf shape within and among hybrids will likely have consequences for fitness. Although differences in leaf and floral traits might show small effects on fitness of plants, we argue that the cumulative effects of many small fitness changes (together with low germination, low survival, and early mortality) would become significant for the hybrids as a whole. This is in line with previous studies that suggest strong cumulative effects of multifarious selection in this system (Wong et al., 2020). Artifically selected families with high seed set for this experiment, genetic background-dependent hybrid breakdown and hybrid fitness on Mount Etna allow us to reconcile these seemingly contradictory observations.

### Potential nucleocytoplasmic incompatibilities/ maternal effects on phenotypes

F_2_ hybrids derived from a reciprocal F_0_ cross showed significant differences in phenotype (Fig. 4; Supplementary Table 3, 4). This indicates potential nucleocytoplasmic incompatibilities and/or maternal effects on later stage phenotypes. While one previous study has shown intrinsic nucleocytoplasmic differences in hybrids of the Mount Etna *Senecio* system (Brennan et al., 2014), this study complements it and shows that nucleocytoplasmic differences could potentially manifest in phenotypes as well, similar to other well-studied systems such as crop hybrids (reviewed in Levin, 2003). However, it is interesting that there was a lack of significant differences in floral traits in the hybrids in this study; this might suggest that nucleocytoplasmic differences or maternal effects would not affect floral developments. However, this should be treated with caution as few floral traits were analysed. So far, only two previous studies have investigated the effect of nucleocytoplasmic incompatibilities (Brennan et al., 2014) and maternal effects (Walter et al., 2021) in this system. Hence more research is needed to study the effects in detail and to distinguish between the extent of hybrid breakdown resulting from nucleocytoplasmic and other incompatibilities, and between nucleocytoplasmic incompatibilities and maternal effects. One future direction would be to analyse species-specific (to *S. aethnensis* and *S. chrysanthemifolius*) nuclear and cytoplasmic genes in *S. squalidus* in the UK. By comparing the distribution of each species’ contribution to the nucleocytoplasmic background of *S. squalidus* through space and time (for example, using herbarium collections available), benefits each cytoplasmic background has on different functions related to survival, fitness and reproduction can be disentangled. This can build on previous work that investigated the proportion of *S. chrysanthemifolius* ancestry in *S. squalidus* samples from different locations (Abbott et al., 2009). Reciprocally backcrossed hybrids could also be used to identify the effect of nucleocytoplasmic interactions.

### Complex leaf architecture and floral characteristics in *Senecio*

Leaf shape in the *Senecio* system presented here is the most defining feature of the two species, yet the adaptive function of (not) having dissection is unknown. The insignificant difference between leaf perimeter:area ratios of greenhouse and wild *S. squalidus* from Exeter, and between F_2_ hybrids and greenhouse *S. squalidus* (Fig. 3) suggest that leaf dissection is not strongly affected by growing conditions and it will continue to be a useful feature to distinguish among *Senecio* species. Greenhouse *S. squalidus* also differed significantly from their wild counterparts overall (Supplementary Table 1) and certain traits (such as leaf length and leaf perimeter; Fig. 3), suggesting some plasticity in leaf shape (mainly size) under different conditions.

The control of leaf shape is also well-known to involve complex interactions among developmental genes that influence cell division and expansion in various parts of a leaf, and also environmental factors (Gonzalez et al., 2012; Tsukaya, 2005; 2006). For example, homeobox genes such as *REDUCED COMPLEXITY* (*RCO*) and *SHOOTMERISTEMLESS* (*STM*) have been found to sculpt leaf shapes (Vlad et al., 2014) and be involved in shape diversity respectively (Kierzkowski et al., 2019). Genes underlying the formation of *Senecio* leaves could be pleiotropic as well as suggested by various studies on other systems (e.g., Jeong et al., 2012; Sayama et al., 2017 on soybean, *Glycine max*). It will require future work to explore the molecular basis of leaf shape evolution in *Senecio* and whether the above studies would apply to this system. What seems certain is that there are likely multiple pleiotropic and/or developmental genes underlying the leaf shape in *Senecio*, as F_2_ hybrids had variable degrees of leaf dissection even within the same family.

Most leaf traits in F_2_ hybrids and *S. squalidus* in this study are correlated (Supplementary Figs. 1, 2). Compared to synthetic hybrid families, *S. squalidus* also had more consistent leaf shape in each population (despite variability among populations; personal field observations). This suggests some selection on *Senecio* leaf shape in the wild. It is interesting that leaf trichomes are also present in the homoploid species, *S. squalidus*, in the UK (Stephen Harris, personal communications) and some of the F_2_ hybrids but not in its parental species *S. aethnensis* and *S. chrysanthemifolius*, and F_1_ hybrids. While evolutionary and ecological implications of this trait are unknown, this suggests that the presence of trichomes in *S. squalidus* did not arise from novel mutations, and it is not necessarily the result of selection for this trait when this species adapted to new conditions in the UK.

In contrast to leaf traits, only one pair of floral traits (number of capitula and apical capitulum pedicel length) in F_2_ hybrids were correlated (Fig. 4), suggesting floral traits might be controlled by more unlinked genes or non-pleiotropic interactions among genes. Besides, ray florets are likely controlled by more than one gene (Kim et al., 2008; Garcês et al., 2016), as the same pair of parents could produce hybrids with both the presence and absence of ray florets. Ray florets have been shown to have significant fitness impact. A variety of *Senecio vulgaris* (rayless), *S. vulgaris var. hibernicus*, was found to possess ray florets which enhanced pollinator attraction (Abbott and Irwin, 1988) and maternal outcrossing (Marshall and Abbott, 1982, 1984). The same benefits were proven for another hybrid species, *Senecio eboracensis*, compared to one of its rayless parental species (Lowe and Abbott, 2004). Future research is needed to understand the genetic and ecological aspects of adaptation of these traits and pinpoint the underlying causes of adaptations.

### Insights into homoploid hybrid speciation

Although hybridisation and hybrid speciation have been well documented, the exact process and conditions favouring the evolution of hybrid generations into stable hybrid population remain unclear. Like Rosenthal et al. (2005), we found that hybrid species phenotypes can be recreated in synthetic lines (e.g., presence of leaf trichomes), revealing the potential of standing variation for evolving new traits and phenotypes that may be adaptive in new environments. In this study, the trait values in synthetic hybrids also started to resemble those of natural hybrids even though they were very early generation hybrids (trait values of F_2_ hybrids are in between those of F_1_ hybrids and *S. squalidus* for leaf area, length, perimeter and width; Supplementary Table 2). While most of the leaf trait values of *S. squalidus* sit between *S. aethnensis* and *S. chrysanthemifolius*’s (length, perimeter, width, perimeter:area, compactness), *S. squalidus* appears to have evolved bigger leaf size (area) and shape (length: width) than both parental species. The effects of these changes and similarities to parental species on the hybrid species’ survival and adaptation require further studies. Future studies could also do more analysis of floral and other traits in comparisons between synthetic and natural hybrids. The ecological significance of ray florets in the hybrid species can also be investigated. Although there are rayless *Senecio* species in the UK (such as *S. vulgaris*, which is self-compatible), species with ray florets were shown to have higher pollination success (discussed above). Given the species is self-incompatible and its common habitat is innutritious substrates such as railway lines and waste grounds (Nevado et al., 2020) where pollinators are scarce, it is suspected that the retainment of ray florets was imperative in attracting available pollinators and subsequent spread of the species.

Wolf et al. (2010) pointed out four potential obstacles hybrids ought to overcome before becoming a ‘species’, including ploidy level, chromosomal rearrangement, genetic incompatibilities and interactions between nuclear genome and endosymbionts. These aspects have been investigated previously (Brennan et al., 2014; 2016; 2019; Chapman et al., 2016; Wong et al., 2020) and in the current study. Firstly, the hybrids created in this study and the homoploid hybrid species *S. squalidus* have the same ploidy level as their progenitors on Mount Etna, and there have been no reports of chromosomal rearrangements. While previous studies reported intrinsic incompatibilities between *S. aethnensis* and *S. chrysanthemifolius* (Brennan et al., 2014; 2016; 2019; Chapman et al., 2016; Wong et al., 2020), this study focused on the last two of the four obstacles—potential nucleocytoplasmic incompatibilities and/or maternal effects, alongside hybrid breakdown that results in low germination, survival, and reproductive success. These likely all work together to limit the number of hybrids on Mount Etna, and thus help maintain the divergence of the two parental species. These lead to one question regarding the colonisation of *S. squalidus*: how did it overcome all the genetic incompatibilities to become a stable population and then spread all over Britain in around 300 years since its introduction to the UK? A study of morphological traits and their change over time, using the historic *S. squalidus* samples preserved in the herbaria, could potentially provide an answer to this interesting question. This study shows some *S. squalidus* traits could be recreated in synthetic hybrids. If these traits had adaptive significance in *S. squalidus*, they would have been ‘ready’ to aid in establishment of the species after the initial hybridisation event. Furthermore, the analysis of DNA from such historical samples could be very informative about the change of genetic composition of the species, revealing how interspecific hybrids evolve into a new species.

Like sunflowers mentioned above, *Senecio* presents another exciting and easy-to-manipulate system for studying hybrid speciation. With the genome assembly of *S. squalidus* on the way, the genetic mechanisms of rapid stabilisation of the hybrid genome and specific selective agents acting on genomic regions contributing to hybrid speciation can be explored in the near future.

## Conclusion

By generating multiple new F_2_ families of hybrids between *S. aethnensis* and *S. chrysanthemifolius*, and analysing four of these families in detail, this study has substantially increased the number of synthetic hybrid lineages studied (only four analysed previously, one in each of Brennan et al., 2014; Chapman et al., 2016; Hegarty et al., 2009; Ross, 2010). These new data allowed us to analyse how hybrid breakdown manifests in crosses between these high- and low-elevation adapted *Senecio* species, and also potential effects of nucleocytoplasmic incompatibilities and/or maternal effects on phenotypes. Further insights are also provided into how the hybrid species*, S. squalidus*, evolved into a stable lineage. Our results reveal that *S. squalidus*’s unique traits could be recreated in synthetic lines and there were signs that synthetic hybrid phenotypes already start to converge with the phenotype of *S. squalidus* as soon as the second generation hybrids. Future research should focus on (a) locating the genes responsible for genetic incompatibilities and maternal effects in hybrids; (b) exploring the complex interplay among developmental and genetic processes that determine leaf shape in this system; and (c) understanding the rapid evolution of *S. squalidus* since its emergence.

### Data archiving

Phenotypic measurements for leaves and floral characters were uploaded to Dryad (DOI: 10.5061/dryad.9p8cz8wm1), accessible through 10.5061/dryad.9p8cz8wm1.

## Supplementary information

Supplementary tables and figures
