# Supplementary material for: Rapid evolution of hybrid breakdown following recent divergence with gene flow in Senecio species on Mount Etna, Sicily
Source: Heredity (Edinb). 2022 Dec 9;130(1):40–52. doi: 10.1038/s41437-022-00576-4 (PMC9814926; doi:10.1038/s41437-022-00576-4)
Supplement: Supplementary file 1 — Supplementary tables and figures [file 41437_2022_576_MOESM1_ESM.docx]

**Supplementary Tables and Figures**

| MANOVA | Test statistic | df1 | df2 | P-value |
| --- | --- | --- | --- | --- |
| Among F_0_, F_1_ and F_2_ | 16.56 | 4.96 | 212.85 | < 0.0001 |
| Among all F_2_ families | 4.61 | 33.04 | 1479.93 | < 0.0001 |
| Between F_2_ families B71 and B87 only | 3.02 | 10.98 | 660.67 | 0.0001 |
|  | | | | |
| ANOSIM | R statistic | | P-value | |
| Between F_2_ hybrids and wild *S. squalidus* | 0.06732 | | 0.0001 | |
| Between F_2_ hybrids and greenhouse grown *S. squalidus* from Exeter | 0.1666 | | 0.0004 | |
| Between wild and greenhouse grown *S. squalidus* from Exeter | 0.04442 | | 0.0012 | |

Supp. Table 1. Differentiation among and between different groups - results of the non-parametric multivariate analysis of variance (MANOVA) and analysis of similarities (ANOSIM).

Supp. Table 2. Summary statistics for the traits measured in each group.

| **Trait**  **(1 mean leaf measurement/individual)** | **B71**  **F_1_ parent 1 (from AC02)**  **(N = 1)** | **B71**  **F_1_ parent 2 (from AC02)**  **(N = 1)** | **B71 (N = 39)** | | | |
| --- | --- | --- | --- | --- | --- | --- |
|  |  |  | **Mean** | **S.D.** | **Min.** | **Max.** |
| Plant height (cm) |  |  | 37.52 | 6.04 | 25.4 | 52 |
| No. effective branches |  |  | 7.13 | 1.65 | 4 | 11 |
| Stem diameter (mm) |  |  | 4.41 | 0.43 | 3.5 | 5.5 |
| No. ray florets |  |  | 11.46 | 2.7 | 0 | 13 |
| No. capitula |  |  | 4.63 | 1.6 | 2 | 9 |
| Apical capitulum disc diameter (mm) |  |  | 6.16 | 0.5 | 5 | 7 |
| Apical capitulum pedicel length (mm) |  |  | 19.64 | 6.53 | 6 | 40 |
| Leaf area (cm^2^) | 69.26 | 11.19 | 54.29 | 69.6 | 6.17 | 360.4 |
| Leaf length (cm) | 59.77 | 57.75 | 37.16 | 17.07 | 15.98 | 85.01 |
| Leaf perimeter (cm) | 128.17 | 141.69 | 82.3 | 40.19 | 32.65 | 199.7 |
| Leaf width (cm) | 2.07 | 2.65 | 3.05 | 2.61 | 0.67 | 11.48 |
| Leaf length:width | 31.49 | 29.05 | 22.76 | 16.63 | 5.65 | 85.73 |
| Leaf perimeter:area | 2.34 | 20.97 | 3.39 | 1.96 | 0.55 | 9.82 |
| Leaf compactness | 314.23 | 2899.69 | 251.6 | 184.7 | 40.42 | 1119 |

| **Trait**  **(1 mean leaf measurement/individual)** | **B87**  **F_1_ parent 1 (from AC03)**  **(N = 1)** | **B87**  **F_1_ parent 2 (from AC03)**  **(N = 1)** | **B87 (N = 29)** | | | |
| --- | --- | --- | --- | --- | --- | --- |
|  |  |  | **Mean** | **S.D.** | **Min.** | **Max.** |
| Plant height (cm) |  |  | 31.93 | 6 | 24.2 | 50 |
| No. effective branches |  |  | 6.72 | 1.6 | 4 | 10 |
| Stem diameter (mm) |  |  | 4.62 | 0.55 | 4 | 5.5 |
| No. ray florets |  |  | 11.28 | 2.05 | 6 | 13 |
| No. capitula |  |  | 5.45 | 1.43 | 3 | 8 |
| Apical capitulum disc diameter (mm) |  |  | 6.09 | 0.49 | 5 | 7 |
| Apical capitulum pedicel length (mm) |  |  | 20.89 | 7.76 | 5 | 39 |
| Leaf area (cm^2^) | 35.48 | 375.17 | 71.58 | 56.03 | 12.84 | 255 |
| Leaf length (cm) | 49.53 | 87.29 | 40.54 | 13.44 | 17.87 | 65.85 |
| Leaf perimeter (cm) | 117.09 | 200.38 | 95.01 | 36.16 | 36.17 | 182.4 |
| Leaf width (cm) | 4.19 | 8.84 | 4.57 | 3.07 | 1.14 | 12.44 |
| Leaf length:width | 14.32 | 9.81 | 13.11 | 5.72 | 3.94 | 25.09 |
| Leaf perimeter:area | 4.07 | 0.53 | 2.6 | 1.64 | 0.88 | 7.72 |
| Leaf compactness | 472.16 | 109.51 | 242.6 | 205.4 | 73.36 | 1065 |

Supp. Table 2 (continued).

| **Trait**  **(1 mean leaf measurement/individual)** | **193B F_1_ parent 1 (from AC15)**  **(N = 1)** | **193B F_1_ parent 2 (from AC15)**  **(N = 1)** | **193B (N = 39)** | | | |
| --- | --- | --- | --- | --- | --- | --- |
|  |  |  | **Mean** | **S.D.** | **Min.** | **Max.** |
| Plant height (cm) |  |  | 39.25 | 5.34 | 25 | 48.9 |
| No. effective branches |  |  | 8.74 | 2.15 | 4 | 13 |
| Stem diameter (mm) |  |  | 5.18 | 0.54 | 4 | 7 |
| No. ray florets |  |  | 12.95 | 0.51 | 11 | 15 |
| No. capitula |  |  | 5.79 | 1.76 | 3 | 11 |
| Apical capitulum disc diameter (mm) |  |  | 5.88 | 0.48 | 5 | 7 |
| Apical capitulum pedicel length (mm) |  |  | 19.74 | 7.6 | 11 | 48 |
| Leaf area (cm^2^) | 38.25 | 159.19 | 91.7 | 68.81 | 8.88 | 250.3 |
| Leaf length (cm) | 40.14 | 59.96 | 45.44 | 13.4 | 17.3 | 73.35 |
| Leaf perimeter (cm) | 81.13 | 126.49 | 101.9 | 35.41 | 37.18 | 184.1 |
| Leaf width (cm) | 1.36 | 4.41 | 4.47 | 3.38 | 0.58 | 12.49 |
| Leaf length:width | 33.63 | 16.13 | 21.04 | 16.09 | 4.05 | 77.51 |
| Leaf perimeter:area | 2.42 | 1.04 | 2.56 | 1.68 | 0.65 | 6.26 |
| Leaf compactness | 194.09 | 122.52 | 229.4 | 129.4 | 71.03 | 607.5 |

| **Trait**  **(1 mean leaf measurement/individual)** | **B228 F_1_ parent 1 (from AC20)**  **(N = 1)** | **B228 F_1_ parent 2 (from AC20)**  **(N = 1)** | **B228 (N = 40)** | | | |
| --- | --- | --- | --- | --- | --- | --- |
|  |  |  | **Mean** | **S.D.** | **Min.** | **Max.** |
| Plant height (cm) |  |  | 38.85 | 5.98 | 27.9 | 54 |
| No. effective branches |  |  | 7.18 | 2.22 | 2 | 12 |
| Stem diameter (mm) |  |  | 4.64 | 0.62 | 3.5 | 6 |
| No. ray florets |  |  | 12.45 | 1.34 | 6 | 13 |
| No. capitula |  |  | 4.69 | 1.34 | 3 | 7 |
| Apical capitulum disc diameter (mm) |  |  | 5.73 | 0.51 | 5 | 7 |
| Apical capitulum pedicel length (mm) |  |  | 16.21 | 6.07 | 7 | 37 |
| Leaf area (cm^2^) | 145.85 | 130.11 | 83.99 | 65.11 | 10.06 | 231.4 |
| Leaf length (cm) | 65.08 | 85.24 | 46.11 | 15.09 | 14.1 | 74.96 |
| Leaf perimeter (cm) | 137.77 | 184.65 | 107 | 43.32 | 29.38 | 201.8 |
| Leaf width (cm) | 3.63 | 3.97 | 4.35 | 3.47 | 0.68 | 14.7 |
| Leaf length:width | 19.14 | 22.42 | 19.97 | 16.23 | 3.71 | 80.91 |
| Leaf perimeter:area | 1.08 | 2.72 | 2.74 | 1.6 | 0.9 | 6.41 |
| Leaf compactness | 150.81 | 507.30 | 266.2 | 169.4 | 42.05 | 824.3 |

Supp. Table 2 (continued).

| **Trait**  **(>1 leaf measurements/individual)** | **All F_0_ *S. aethnensis***  **(N = 7)** | | | | **All F_0_ *S. chrysanthemifolius***  **(N = 6)** | | | | **All F_1_ individuals**  **(N = 21)** | | | |
| --- | --- | --- | --- | --- | --- | --- | --- | --- | --- | --- | --- | --- |
|  | **Mean** | **S.D.** | **Min.** | **Max.** | **Mean** | **S.D.** | **Min.** | **Max.** | **Mean** | **S.D.** | **Min.** | **Max.** |
| Leaf area (cm^2^) | 4.45 | 1.28 | 3.05 | 7.03 | 120.89 | 82.32 | 39.52 | 258.96 | 119.38 | 111.40 | 20.31 | 393.04 |
| Leaf length (cm) | 10.04 | 1.66 | 8.03 | 12.08 | 50.87 | 16.27 | 22.2 | 68.46 | 62.39 | 17.33 | 38.87 | 102.89 |
| Leaf perimeter (cm) | 20.43 | 3.52 | 16.83 | 24.97 | 227.9 | 87.84 | 60.5 | 304.1 | 136.19 | 39.73 | 78.06 | 240.45 |
| Leaf width (cm) | 0.68 | 0.16 | 0.46 | 0.96 | 25.25 | 12.46 | 4.97 | 40.73 | 3.81 | 2.27 | 0.82 | 9.39 |
| Leaf length:width | 15.53 | 5.06 | 11.49 | 25.23 | 2.45 | 1.11 | 1.18 | 4.47 | 21.30 | 10.89 | 8.13 | 47.66 |
| Leaf perimeter:area | 4.82 | 1.04 | 3.30 | 6.53 | 2.73 | 2.57 | 1.04 | 7.70 | 2.29 | 1.88 | 0.45 | 6.01 |
| Leaf compactness | 99.98 | 31.23 | 70.98 | 154.72 | 713.26 | 829.00 | 62.93 | 2339.91 | 300.19 | 277.94 | 71.93 | 1112.2 |

| **Trait**  **(>1 leaf measurements/individual)** | **All F_2_ individuals**  **(N = 379)** | | | | **Wild *S. squalidus***  **(N = 583)** | | | | **Greenhouse *S. squalidus***  **(N = 124)** | | | |
| --- | --- | --- | --- | --- | --- | --- | --- | --- | --- | --- | --- | --- |
|  | **Mean** | **S.D.** | **Min.** | **Max.** | **Mean** | **S.D.** | **Min.** | **Max.** | **Mean** | **S.D.** | **Min.** | **Max.** |
| Leaf area (cm^2^) | 77.9 | 81.61 | 6.07 | 387.3 | 67.64 | 70.92 | 5.05 | 364.97 | 56.64 | 61.71 | 5.2 | 368.2 |
| Leaf length (cm) | 43.35 | 15.28 | 12.82 | 87.02 | 27.68 | 14.42 | 4.86 | 81.36 | 26.91 | 13.05 | 5.21 | 68.27 |
| Leaf perimeter (cm) | 99.03 | 40.43 | 26.92 | 230 | 72.64 | 45.97 | 11.7 | 295.1 | 67.22 | 37.54 | 15.33 | 220.6 |
| Leaf width (cm) | 4.2 | 3.59 | 0.28 | 20.57 | 6.13 | 4.81 | 0.40 | 29.63 | 5.05 | 4.03 | 0.41 | 23.6 |
| Leaf length:width | 19.15 | 20.08 | 2 | 183.9 | 6.86 | 6.58 | 0.95 | 58.58 | 9.36 | 10.28 | 0.97 | 70.01 |
| Leaf perimeter:area | 2.79 | 2.73 | 0.33 | 21.08 | 2.11 | 2.44 | 0.31 | 22.2 | 2.28 | 2.19 | 0.37 | 16.32 |
| Leaf compactness | 253.2 | 289.6 | 30.29 | 2931 | 159.17 | 302.16 | 15.98 | 3031 | 152.8 | 235.7 | 21.61 | 2251 |

Supp. Table 3. Results of the Kruskal-Wallis test. *indicates significant P-values.

| **Trait** | **Among four F_2_ families**  **(df = 3)** | | **All F_2_ and *S. squalidus***  **(df = 1)** | | **All F2 and *S. squalidus* Exeter/ greenhouse**  **(df = 1)** | | ***S. squalidus* Exeter/**  **field and greenhouse**  **(df = 1)** | |
| --- | --- | --- | --- | --- | --- | --- | --- | --- |
|  | **χ^2^** | **P - value** | **χ^2^** | **P - value** | **χ^2^** | **P - value** | **χ^2^** | **P - value** |
| Presence of trichomes | 17.91 | 0.00046* |  |  |  |  |  |  |
| Plant height | 27.67 | 4.25 x 10^-06^* |  |  |  |  |  |  |
| No. effective branches | 19.45 | 0.00022* |  |  |  |  |  |  |
| Stem diameter | 34.28 | 1.73 x 10^-07^* |  |  |  |  |  |  |
| No. ray florets | 27.04 | 5.76 x 10^-06^* |  |  |  |  |  |  |
| No. capitula | 12.49 | 0.0059* |  |  |  |  |  |  |
| Apical capitulum disc diameter | 14.69 | 0.0021* |  |  |  |  |  |  |
| Apical capitulum pedicel length | 11.71 | 0.0084* |  |  |  |  |  |  |
| Leaf length | 14.36 | 0.0025* | 234.42 | < 2.2 x 10^-16^* | 96.88 | < 2.2 x 10^-16^* | 22.92 | 1.69 x 10^-06^* |
| Leaf width | 8.90 | 0.031* | 48.03 | 4.20 x 10^-12^* | 5.73 | 0.017* | 0.032 | 0.86 |
| Leaf perimeter | 12.52 | 0.0058* | 126.85 | < 2.2 x 10^-16^* | 60.67 | 6.76 x 10^-15^* | 13.80 | 0.00020* |
| Leaf area | 16.15 | 0.0011* | 6.03 | 0.014* | 7.46 | 0.0063* | 3.76 | 0.052 |
| Leaf length:width | 6.59 | 0.086 | 310.19 | < 2.2 x 10^-16^* | 76.39 | < 2.2 x 10^-16^* | 13.94 | 0.00019* |
| Leaf perimeter:area | 6.23 | 0.10 | 22.11 | 2.58 x 10^-06^* | 2.53 | 0.11 | 0.00011 | 0.99 |
| Leaf compactness | 2.28 | 0.52 | 161.2 | < 2.2 x 10^-16^* | 48.94 | 2.65 x 10^-12^* | 7.01 | 0.0081* |

| **Presence of trichomes** | | | |  | **Leaf length** | | | |
| --- | --- | --- | --- | --- | --- | --- | --- | --- |
|  | B71 | B87 | 193B |  |  | B71 | B87 | 193B |
| B87 | 1 | - | - |  | B87 | 0.4663 | - | - |
| 193B | 0.0486* | 0.0033* | - |  | 193B | 0.0045* | 1 | - |
| B228 | 1 | 1 | 0.0074* |  | B228 | 0.0062* | 1 | 1 |
|  |  |  |  |  |  |  |  |  |
| **Plant height** | | | |  | **Leaf width** | | | |
|  | B71 | B87 | 193B |  |  | B71 | B87 | 193B |
| B87 | 0.0015* | - | - |  | B87 | 0.019* | - | - |
| 193B | 0.8278 | 2.20 x 10^-05^* | - |  | 193B | 0.2 | 1 | - |
| B228 | 1 | 5.30 x 10^-05^* | 1 |  | B228 | 0.179 | 1 | 1 |
|  |  |  |  |  |  |  |  |  |
| **No. of effective branches** | | | |  | **Leaf perimeter** | | | |
|  | B71 | B87 | 193B |  |  | B71 | B87 | 193B |
| B87 | 1 | - | - |  | B87 | 0.341 | - | - |
| 193B | 0.00619* | 0.00032* | - |  | 193B | 0.011* | 1 | - |
| B228 | 1 | 1 | 0.02256* |  | B228 | 0.01* | 1 | 1 |
|  |  |  |  |  |  |  |  |  |
| **Stem diameter** | | | |  | **Leaf area** | | | |
|  | B71 | B87 | 193B |  |  | B71 | B87 | 193B |
| B87 | 0.6599 | - | - |  | B87 | 0.0197* | - | - |
| 193B | 2.50 x 10^-08^* | 0.001* | - |  | 193B | 0.0051* | 1 | - |
| B228 | 0.3003 | 1 | 0.0034* |  | B228 | 0.0033* | 1 | 1 |
|  |  |  |  |  |  |  |  |  |
| **No. of ray florets** | | | |  | **Leaf length:width** | | | |
|  | B71 | B87 | 193B |  |  | B71 | B87 | 193B |
| B87 | 1 | - | - |  | B87 | 0.025* | - | - |
| 193B | 0.00013* | 3.70 x 10^-05^* | - |  | 193B | 1 | 0.857 | - |
| B228 | 0.10361 | 0.03327* | 0.12789 |  | B228 | 1 | 0.99 | 1 |
|  |  |  |  |  |  |  |  |  |
| **No. of capitula** | | | |  | **Leaf perimeter:area** | | | |
|  | B71 | B87 | 193B |  |  | B71 | B87 | 193B |
| B87 | 0.218 | - | - |  | B87 | 0.11 | - | - |
| 193B | 0.062 | 1 | - |  | 193B | 0.22 | 1 | - |
| B228 | 1 | 0.135 | 0.03* |  | B228 | 1 | 1 | 1 |
|  |  |  |  |  |  |  |  |  |
| **Apical capitulum disc diameter** | | | |  | **Leaf compactness** | | | |
|  | B71 | B87 | 193B |  |  | B71 | B87 | 193B |
| B87 | 1 | - | - |  | B87 | 1 | - | - |
| 193B | 0.127 | 0.5798 | - |  | 193B | 1 | 1 | - |
| B228 | 0.0047* | 0.0407* | 1 |  | B228 | 1 | 0.72 | 1 |
|  |  |  |  |  |  |  |  |  |
| **Apical capitulum pedicel length** | | | |  |  |  |  |  |
|  | B71 | B87 | 193B |  |  |  |  |  |
| B87 | 1 | - | - |  |  |  |  |  |
| 193B | 1 | 1 | - |  |  |  |  |  |
| B228 | 0.032* | 0.039* | 0.091 |  |  |  |  |  |

Supp. Table 4. P-values for pairwise Wilcoxon rank-sum tests, Bonferroni-corrected, among the four F_2_ families. *indicates significant P-values.

| **F_2_ & wild *S. squalidus* (Fig. 5)** | **PC1** | **PC2** | **PC3** | **PC4** |
| --- | --- | --- | --- | --- |
| Leaf area | 0.486544 | -0.17395 | 0.849429 | -0.10717 |
| Leaf length | 0.506801 | 0.602124 | -0.08998 | 0.610331 |
| Leaf perimeter | 0.553936 | 0.207621 | -0.36545 | -0.71868 |
| Leaf width | 0.446748 | -0.75105 | -0.36989 | 0.315458 |
| Standard deviation | 1.724 | 0.7903 | 0.61993 | 0.13741 |
| Proportion of variance | 0.743 | 0.1562 | 0.09608 | 0.00472 |
| Cumulative proportion | 0.743 | 0.8992 | 0.99528 | 1 |
| **F_2_ & *S. squalidus* Exeter population from the field and greenhouse (Fig. 6)** | **PC1** | **PC2** | **PC3** | **PC4** |
| Leaf area | 0.501922 | 0.218259 | 0.83325 | 0.078314 |
| Leaf length | 0.506037 | -0.57463 | -0.09451 | -0.63623 |
| Leaf perimeter | 0.556378 | -0.24887 | -0.33738 | 0.717412 |
| Leaf width | 0.427136 | 0.748482 | -0.42771 | -0.27275 |
| Standard deviation | 1.717 | 0.8476 | 0.5667 | 0.1111 |
| Proportion of variance | 0.737 | 0.1796 | 0.0803 | 0.00309 |
| Cumulative proportion | 0.737 | 0.9166 | 0.9969 | 1 |

Supp. Table 5. PCA loading values for Figure 5, 6, 7, and Supp. Fig. 3, 4.

| **All F_2_ families (Fig. 7)** | **PC1** | **PC2** | **PC3** | **PC4** | **PC5** | **PC6** | **PC7** | **PC8** | **PC9** | **PC10** | **PC11** | **PC12** |
| --- | --- | --- | --- | --- | --- | --- | --- | --- | --- | --- | --- | --- |
| No. of ray florets | -0.02051 | -0.3478 | 0.269065 | -0.55339 | 0.160023 | -0.59022 | 0.260508 | -0.0545 | -0.21913 | -0.02662 | 0.080376 | 0.001084 |
| Trichome | 0.081807 | 0.35738 | -0.36724 | -0.10445 | -0.36822 | -0.072 | 0.73086 | -0.18278 | 0.095549 | -0.02184 | 0.042316 | 0.008673 |
| No. of effective branches | 0.06975 | -0.37008 | -0.4702 | 0.302701 | 0.165363 | -0.01574 | 0.242783 | 0.527518 | -0.41326 | -0.08783 | 0.044937 | -0.01175 |
| Plant height | 0.084842 | -0.45141 | 0.088214 | -0.25737 | -0.4113 | 0.587944 | 0.042835 | -0.23908 | -0.29136 | -0.23454 | -0.03567 | 0.000115 |
| Disc diameter | -0.09641 | 0.327417 | 0.319651 | -0.29589 | 0.443943 | 0.48348 | 0.338635 | 0.377892 | -0.03719 | -0.062 | -0.01178 | -0.0096 |
| Capitulum length | -0.16677 | -0.03771 | -0.47147 | -0.13159 | 0.58374 | 0.173704 | -0.04035 | -0.57905 | -0.10161 | 0.089476 | 0.07063 | 0.001398 |
| Stem diameter | 0.164533 | -0.52455 | 0.026258 | 0.077045 | 0.173804 | 0.120219 | 0.276093 | 0.018041 | 0.744086 | 0.10878 | -0.06641 | -0.00577 |
| No. of capitulum | 0.099681 | -0.01183 | 0.477012 | 0.627957 | 0.17528 | -0.03477 | 0.344083 | -0.38348 | -0.25481 | -0.07538 | 0.001796 | -0.00773 |
| Leaf area | 0.4753 | 0.080773 | 0.000844 | -0.0314 | 0.07947 | 0.022582 | -0.12127 | -0.00261 | 0.09103 | -0.28654 | 0.805063 | -0.08077 |
| Leaf length | 0.482864 | 0.030854 | 0.027917 | -0.07307 | 0.012886 | 0.077921 | -0.01661 | 0.009403 | -0.1685 | 0.577187 | -0.00972 | 0.625731 |
| Leaf perimeter | 0.500425 | 0.066303 | -0.01585 | -0.09212 | 0.048154 | 0.017151 | -0.0382 | -0.01986 | -0.12912 | 0.320253 | -0.26214 | -0.73758 |
| Leaf width | 0.444091 | 0.121676 | -0.10077 | -0.07099 | 0.186348 | -0.11861 | -0.09918 | -0.0471 | 0.059412 | -0.62363 | -0.51184 | 0.239818 |
| Standard deviation | 1.9183 | 1.3496 | 1.1629 | 1.0194 | 0.98676 | 0.86299 | 0.85091 | 0.79535 | 0.72608 | 0.5786 | 0.40572 | 0.07514 |
| Proportion of variance | 0.3067 | 0.1518 | 0.1127 | 0.0866 | 0.08114 | 0.06206 | 0.06034 | 0.05271 | 0.04393 | 0.0279 | 0.01372 | 0.00047 |
| Cumulative proportion | 0.3067 | 0.4584 | 0.5711 | 0.6577 | 0.73887 | 0.80093 | 0.86127 | 0.91398 | 0.95791 | 0.9858 | 0.99953 | 1 |

Supp. Table 5 (continued).

| **F_2_ and parents in the previous two generations (Supp. Fig. 3)** | **PC1** | **PC2** | **PC3** | **PC4** |
| --- | --- | --- | --- | --- |
| Leaf area | 0.478548 | -0.17745 | 0.854647 | -0.09531 |
| Leaf length | 0.497987 | 0.65902 | -0.07979 | 0.557974 |
| Leaf perimeter | 0.550817 | 0.164264 | -0.35646 | -0.73658 |
| Leaf width | 0.468616 | -0.7122 | -0.36899 | 0.370169 |
| Standard deviation | 1.7505 | 0.7335 | 0.62043 | 0.11218 |
| Proportion of variance | 0.7661 | 0.1345 | 0.09623 | 0.00315 |
| Cumulative proportion | 0.7661 | 0.9006 | 0.99685 | 1 |
| **B71 (Supp. Fig. 4a)** | **PC1** | **PC2** | **PC3** | **PC4** |
| Leaf area | -0.50553 | -0.35264 | -0.7789 | 0.115725 |
| Leaf length | -0.50789 | 0.536124 | -0.01325 | -0.67413 |
| Leaf perimeter | -0.53481 | 0.381303 | 0.278582 | 0.700695 |
| Leaf width | -0.44773 | -0.66545 | 0.561716 | -0.20294 |
| Standard deviation | 1.748 | 0.8499 | 0.4612 | 0.09414 |
| Proportion of variance | 0.764 | 0.1806 | 0.05318 | 0.00222 |
| Cumulative proportion | 0.764 | 0.9446 | 0.99778 | 1 |
| **B87 (Supp. Fig. 4b)** | **PC1** | **PC2** | **PC3** | **PC4** |
| Leaf area | 0.493633 | -0.04243 | -0.86209 | -0.10642 |
| Leaf length | 0.503233 | 0.59629 | 0.185051 | 0.597453 |
| Leaf perimeter | 0.542972 | 0.1481 | 0.393357 | -0.72699 |
| Leaf width | 0.456359 | -0.78785 | 0.260432 | 0.32126 |
| Standard deviation | 1.7835 | 0.7138 | 0.54884 | 0.09133 |
| Proportion of variance | 0.7952 | 0.1274 | 0.07531 | 0.00209 |
| Cumulative proportion | 0.7952 | 0.9226 | 0.99791 | 1 |

Supp. Table 5 (continued).

Supp. Table 5 (continued).

| **193B (Supp. Fig. 4c)** | **PC1** | **PC2** | **PC3** | **PC4** |
| --- | --- | --- | --- | --- |
| Leaf area | 0.465634 | 0.664021 | 0.569753 | -0.13282 |
| Leaf length | 0.513262 | -0.51258 | 0.319998 | 0.609447 |
| Leaf perimeter | 0.533716 | -0.41077 | -0.12721 | -0.72817 |
| Leaf width | 0.484658 | 0.357222 | -0.74619 | 0.284073 |
| Standard deviation | 1.7916 | 0.6627 | 0.5827 | 0.10727 |
| Proportion of variance | 0.8024 | 0.1098 | 0.0849 | 0.00288 |
| Cumulative proportion | 0.8024 | 0.9122 | 0.9971 | 1 |
| **B228 (Supp. Fig. 4d)** | **PC1** | **PC2** | **PC3** | **PC4** |
| Leaf area | -0.48045 | -0.40539 | 0.776635 | 0.040839 |
| Leaf length | -0.50142 | 0.611633 | 0.041169 | -0.61056 |
| Leaf perimeter | -0.54705 | 0.311358 | -0.21516 | 0.746659 |
| Leaf width | -0.46742 | -0.60384 | -0.59063 | -0.26086 |
| Standard deviation | 1.7549 | 0.7684 | 0.5643 | 0.10684 |
| Proportion of variance | 0.7699 | 0.1476 | 0.0796 | 0.00285 |
| Cumulative proportion | 0.7699 | 0.9175 | 0.9971 | 1 |


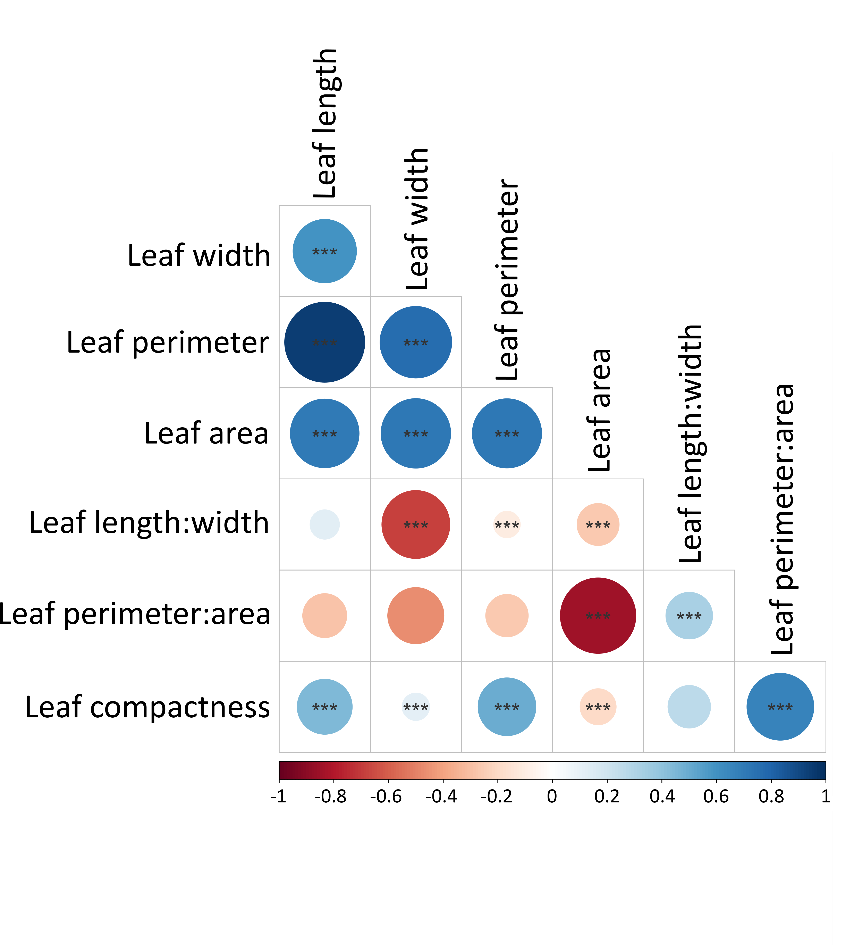

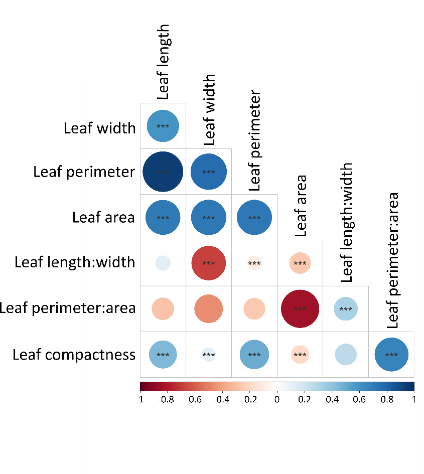


**Supp. Figure 1.** Spearman’s correlation between all pairs of traits measured in all wild *S. squalidus* individuals. Size and colour of circles represent Spearman’s Rho (see scale bar); asterisks in circles show significance: *: p-value < 0.05, **: p-value < 0.01, ***: p-value < 0.01.


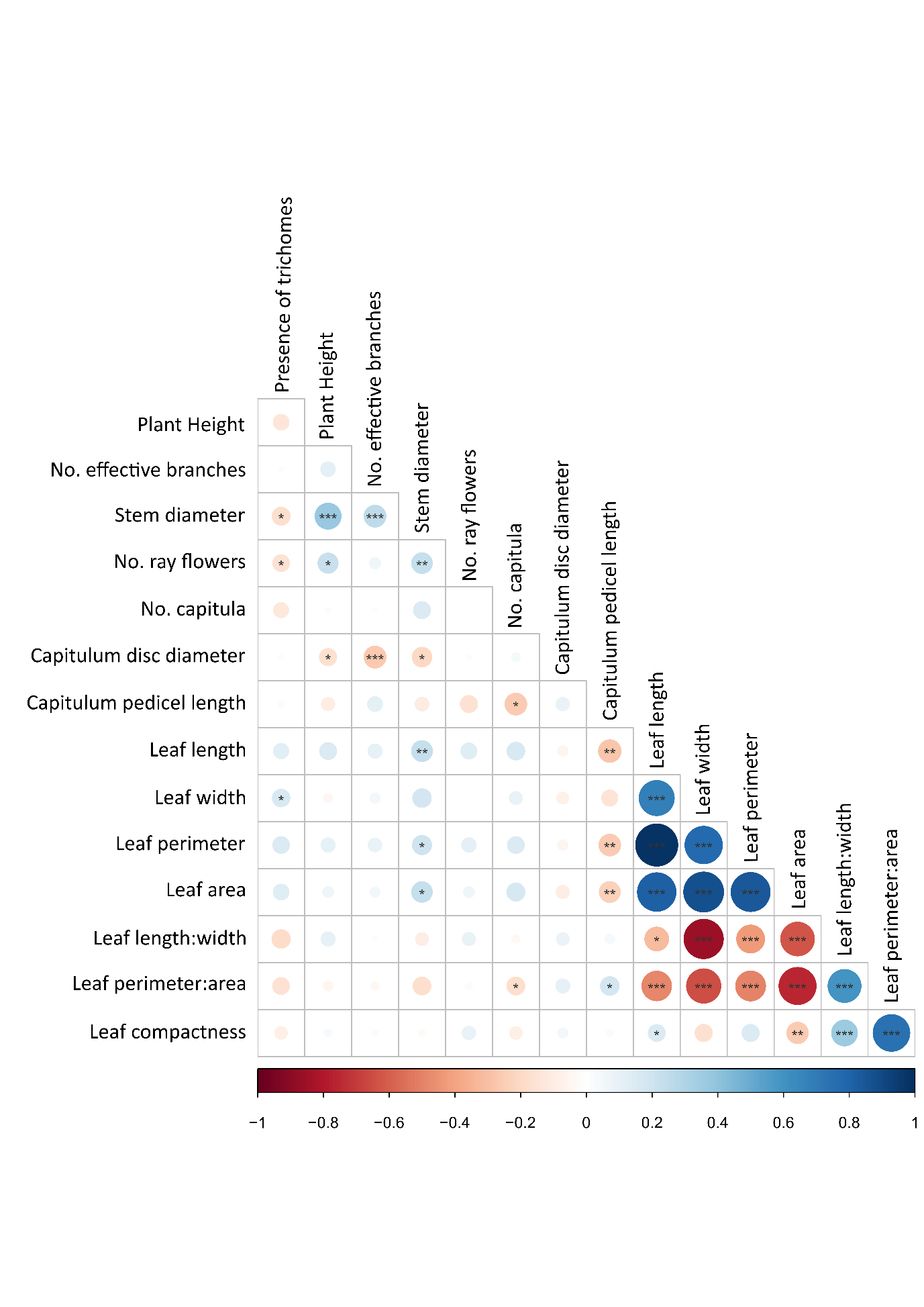

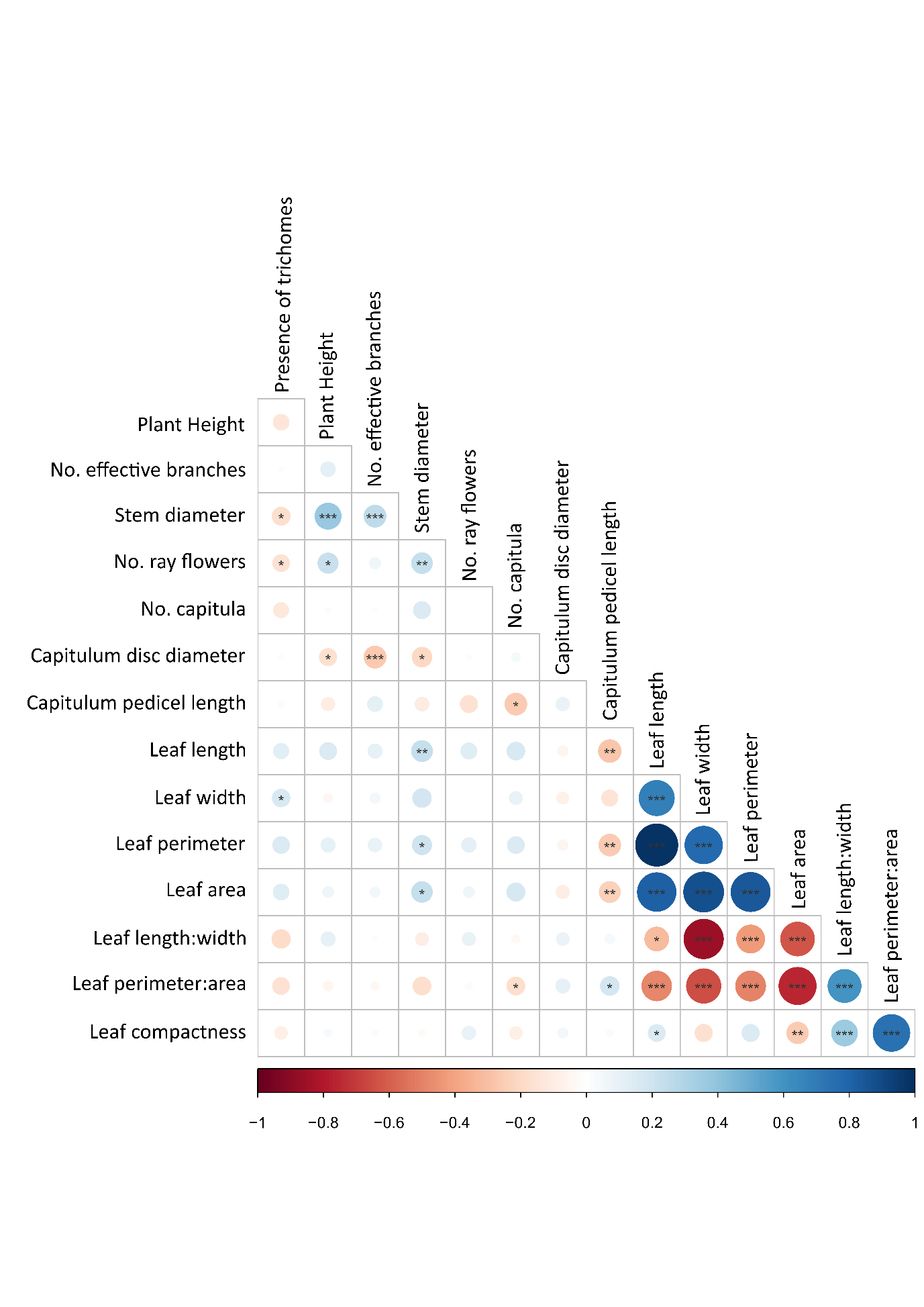


**Supp. Figure 2.** Spearman’s correlation between all pairs of traits measured in all F_2_ individuals. Size and colour of circles represent Spearman’s Rho (see scale bar); asterisks in circles show significance: *: p-value < 0.05, **: p-value < 0.01, ***: p-value < 0.01.


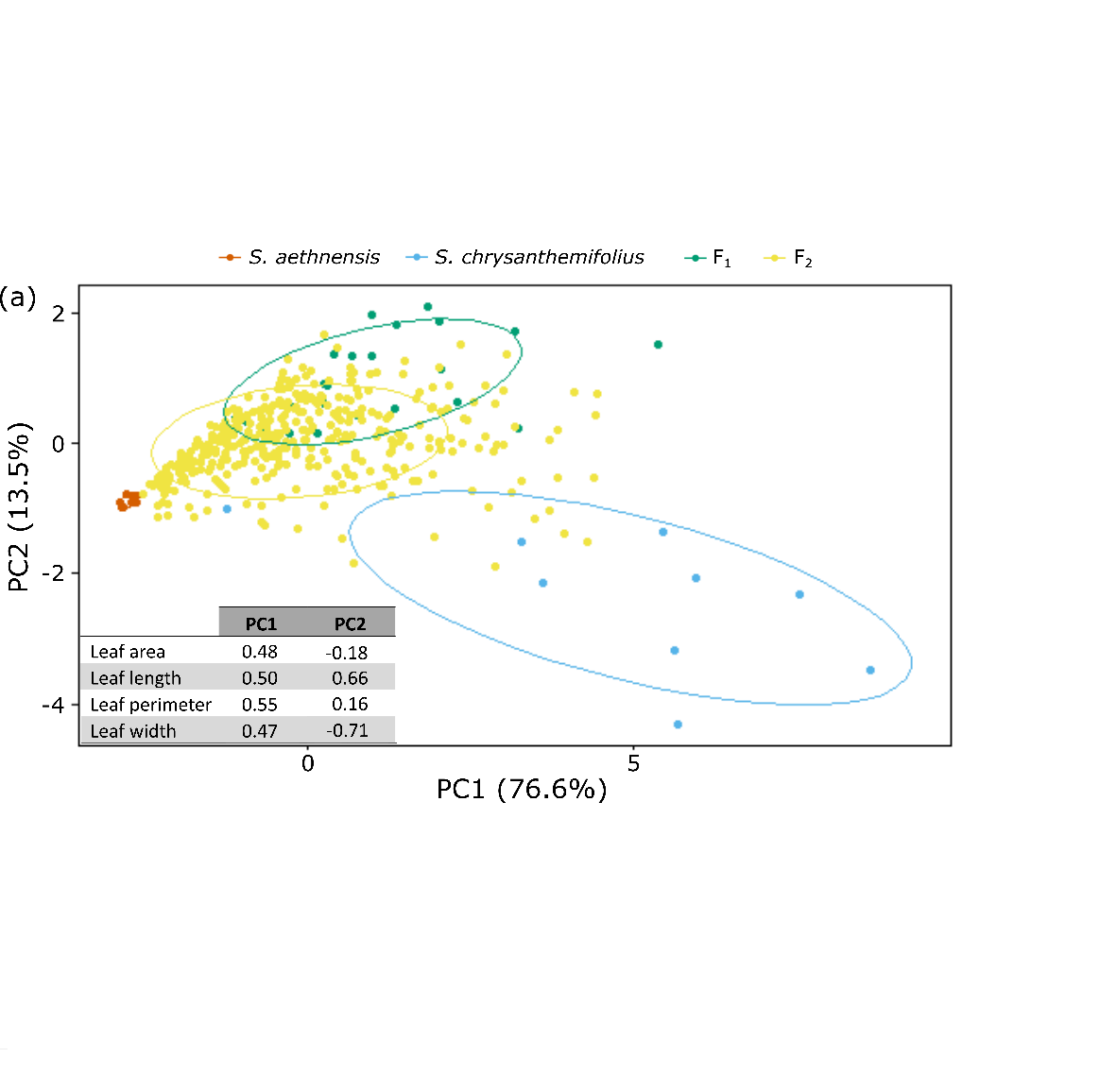


**Supp. Figure 3.** Ordination plot (PCA) of all F_2_ individuals used in this study, and their respective parents in the previous two generations. Ellipses represent 95% CI of the data. Full tables of loading values are in Supp. Table 5.


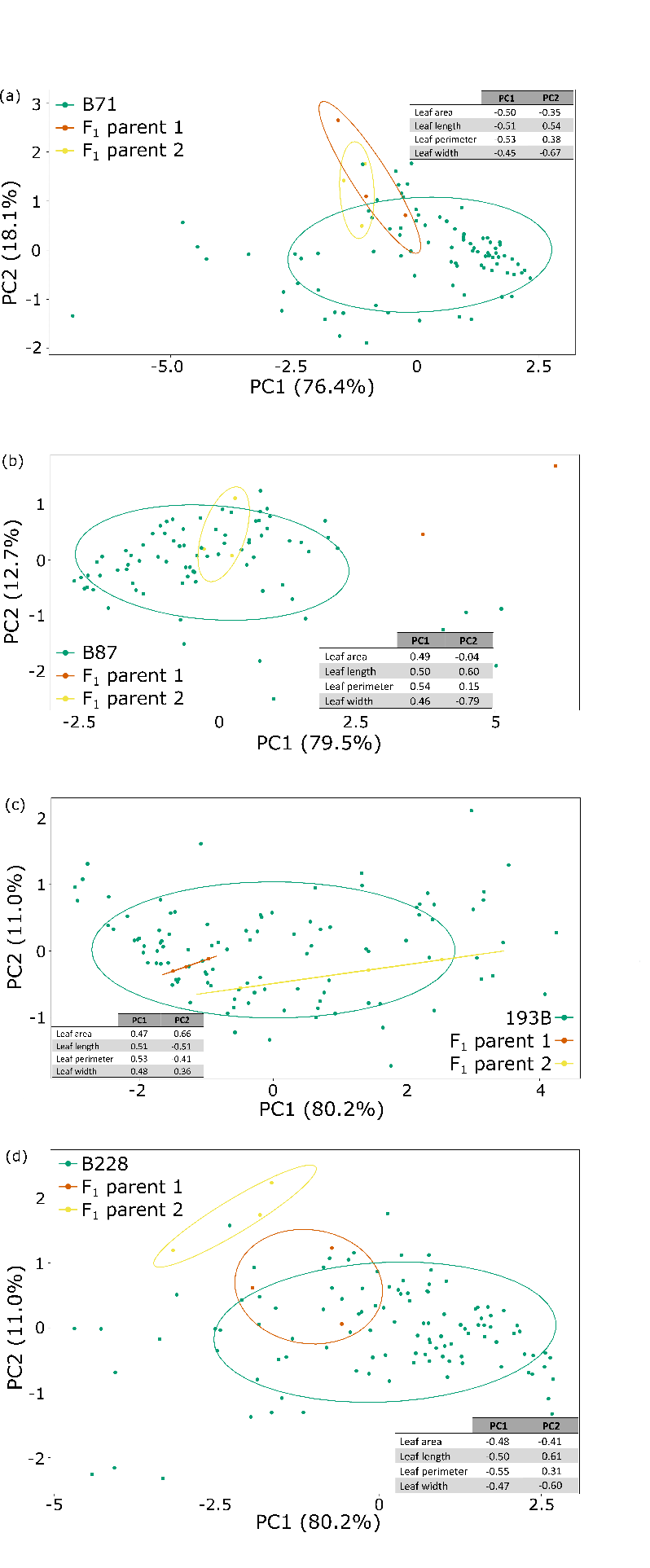


**Supp. Figure 4.** PCA of each F_2_ family with their respective F_1_ parents. Ellipses represent 95% CI of the data. Loading values for PC1 and PC2 are embedded in the plot. Full tables of loading values are in Supp. Table 5.
